# Decoupling Triplet Population from Intersystem Crossing in TADF Materials

**DOI:** 10.1021/jacs.6c10012

**Published:** 2026-09-08

**Authors:** Erin M. Holdsworth, Oliver Millington, Victor Gray, Tarig B. E. Mustafa, Sebastian Gorgon, Lars van Turnhout, Weixuan Zeng, Alexander J. Gillett, Akshay Rao, Daniel G. Congrave, Hugo Bronstein

**Affiliations:** † Department of Chemistry, 2152 University of Cambridge, Cambridge CB2 1EW, U.K.; ‡ Cavendish Laboratory, University of Cambridge, Cambridge CB3 0HE, U.K.; § Department of Chemistry, Ångström Laboratory, 8097 Uppsala University, SE-751 20 Uppsala, Sweden; ∥ Zhangjiang Laboratory, Shanghai 201210, China; ⊥ Department of Physics, Chemistry and Biology (IFM), 4566 Linköping University, 581 83 Linköping, Sweden; # Chemistry Research Laboratory, 6396 University of Oxford, Oxford OX1 3TA, U.K.

## Abstract

The characterization of thermally activated delayed fluorescence (TADF) relies on detecting delayed emission following triplet formation. However, triplet population under optical excitation is fundamentally limited by the intrinsic intersystem crossing (ISC) efficiency of the emitter and in direct competition with often desirable singlet radiative decay. Here, we implement a general optical strategy to directly populate the triplet states of TADF chromophores in solution via diffusional triplet energy transfer, circumventing reliance on intrinsic ISC. This approach can enhance delayed-fluorescence detectability by up to 3 orders of magnitude across structurally diverse emitters spanning the blue to near-infrared and disparate emission time scales, converting near-threshold experiments into routine measurements. Through decoupling triplet population from ISC, we reveal previously hidden intrinsic TADF in two state-of-the-art chromophores, leading to a striking conclusion: unimolecular TADF remains highly relevant within multiple high-performing material families where delayed fluorescence—and the resulting high device performance—has previously been attributed solely to intermolecular solid-state effects. Accordingly, through providing an approach to directly interrogate the intrinsic triplet state photophysics of TADF materials, triplet sensitization can enable the identification and understanding of previously inaccessible photophysical behavior and property combinations, providing a foundation for future advances in TADF materials research.

## Graphical abstract

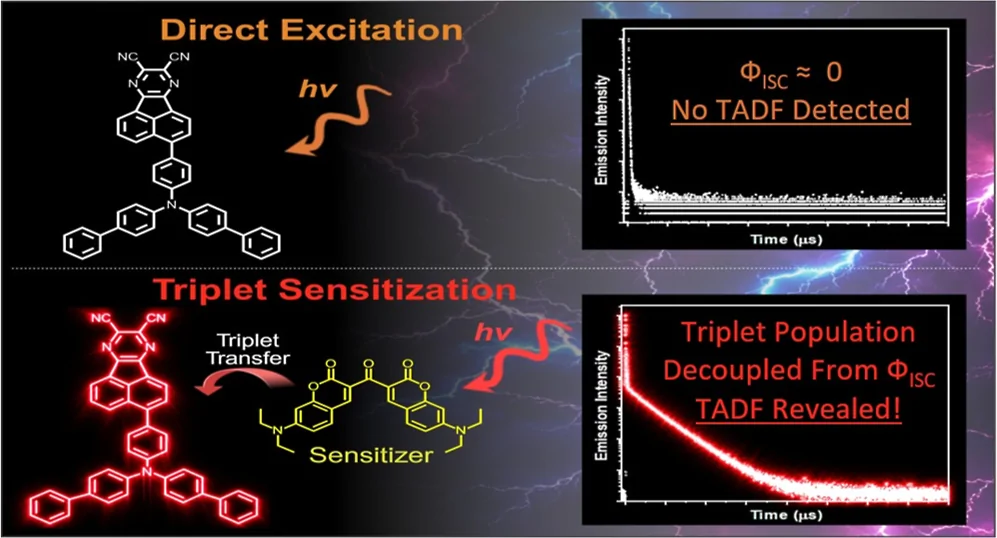

## Introduction

Molecules exhibiting thermally activated delayed fluorescence (TADF) offer unique opportunities for the interconversion of singlet (S) and triplet (T) excited states,^1^ becoming central in applications including next-generation organic light emitting diodes (OLEDs), organic photovoltaics (OPVs),^2^ imaging,^3–5^ sensing^6,7^ and photocatalysis.^8–11^

TADF behavior is conventionally characterized through photophysical experiments in which optical excitation populates the singlet state (Figure 1 a).^12–15^ Singlet radiative decay (rate constant = *k*_r_^S^) leads to a nanosecond prompt photoluminescence (PL) decay component. If the rate of intersystem crossing (ISC, rate constant = *k*_ISC_) is sufficient, triplets are generated, which can lead to micro–millisecond delayed PL after reverse intersystem crossing (RISC, rate constant = *k*_RISC_). Central TADF parameters such as *k*_ISC_ and *k*_RISC_ are extracted through considering the relative amplitudes and lifetimes of prompt and delayed fluorescence components.^16^ However, a fundamental limitation of this approach is that triplet formation under direct optical excitation is intrinsically coupled to *k*_ISC_ and therefore competes directly with singlet radiative decay.

**Figure 1:**
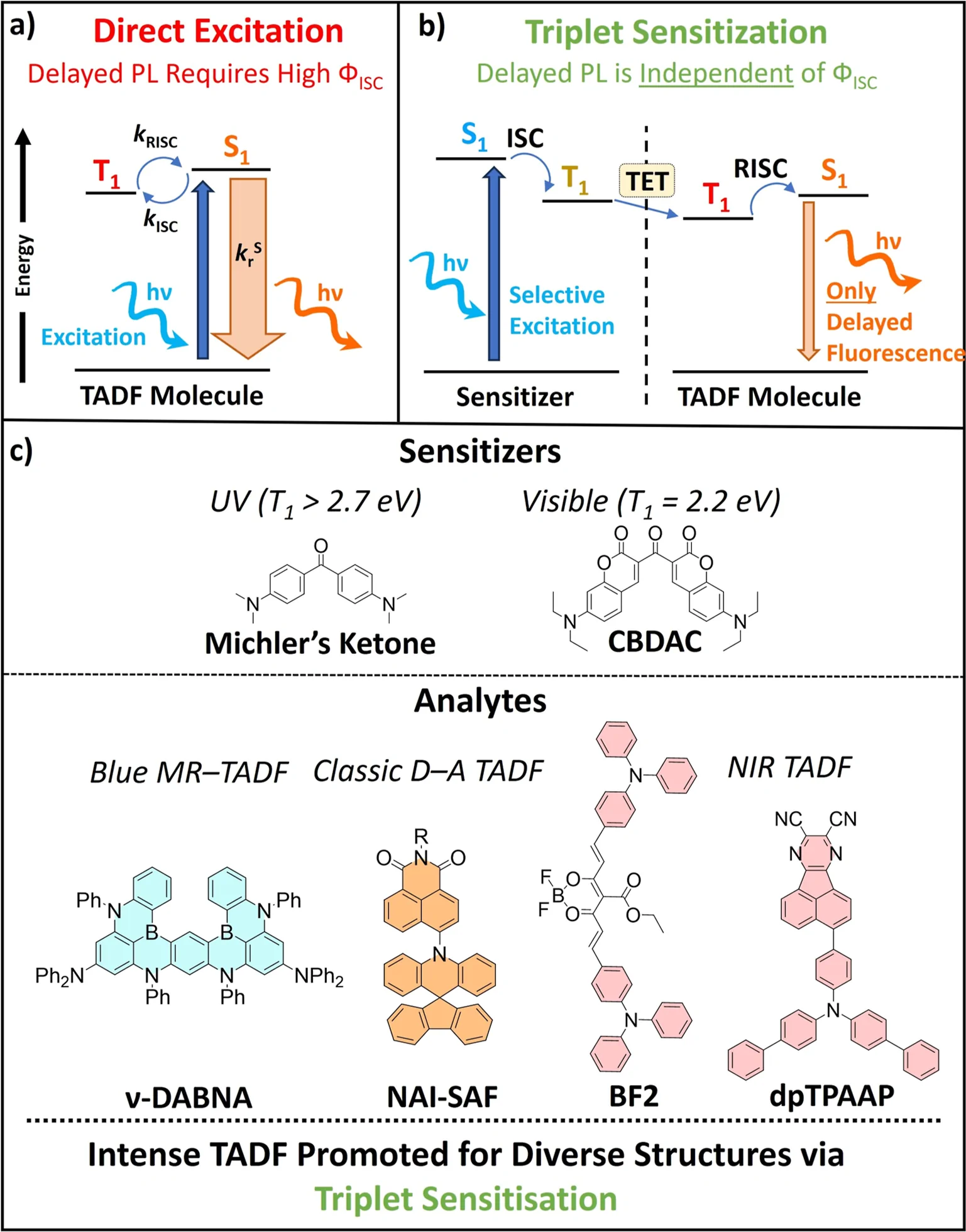
(A) Jablonski diagram for direct excitation experiments; (b) Jablonski diagram for sensitization experiments–delayed PL is enabled independent of intrinsic intersystem crossing efficiency; (c) Structures of the investigated sensitizers and analytes.

This limitation has become increasingly acute as contemporary TADF materials expand toward higher radiative rates. For blue OLEDs, reducing the triplet exciton residence time of TADF materials through accelerating radiative decay is widely regarded as essential to achieving long device lifetimes.^17–25^ Analogously, enhancing the radiative rate of near-infrared (NIR) TADF is key to mitigate nonradiative losses imposed by the energy gap law to facilitate efficient OPV and sensing applications.^26–31^

As the radiative rates of TADF molecules are increased, intrinsic ISC yields are inevitably suppressed (Φ_ISC_ → 0), rendering delayed fluorescence progressively more difficult—or even impossible in principle—to observe under direct excitation.^32–34^ Particularly, Zysman-Colman and Samuel et al. recently showed that the ideal TADF OLED emitter should satisfy *k*_r_^S^ + *k*_RISC_ ≫ *k*_ISC_ i.e. any population of the TADF triplet state through intrinsic ISC should be avoided.^17^ In this regime, the absence of observable delayed fluorescence under direct excitation does not necessitate the absence of viable RISC pathways, but rather reflects a methodological blind spot in conventional photophysical characterization which could present a substantial complication for the analysis of new, high-performing materials.

The analysis of TADF materials can be further complicated in the solid state, where delayed fluorescence dynamics are often multicomponent and sensitive to factors such as aggregation, intermolecular charge–transfer interactions, host environment, and morphological heterogeneity.^35,36^ While these effects can influence parameters such as *k*_ISC_ and *k*_RISC_ and even enable or enhance TADF in films and devices, they can also complicate mechanistic interpretation. Understanding the intrinsic triplet-state properties of TADF chromophores therefore provides a vital foundation for disentangling intrinsic molecular behavior from the additional complexities introduced by the solid-state environment, thereby providing the fundamental understanding upon which future molecular design principles can be built.

In light of these considerations, there is a clear need for experimental approaches that enable optical access to the triplet manifolds of TADF chromophores independent of their intrinsic ISC efficiency. Such strategies will become particularly appealing for establishing the intrinsic triplet-state properties of emerging high radiative rate TADF materials, which in turn provide a crucial anchor point for interpreting the complex, multicomponent delayed fluorescence dynamics commonly observed in the solid state. In this work, we introduce a simple triplet sensitization approach that decouples triplet population from ISC (Figure 1b), providing direct experimental access to the intrinsic triplet state photophysics of diverse TADF materials (Figure 1c) and enabling delayed fluorescence to be interrogated independently of intrinsic ISC efficiency.

## Results and Discussion

To establish triplet sensitization as a viable strategy for interrogating TADF photophysics, we first apply the approach to the donor–acceptor (D–A) TADF emitter **NAI-SAF-4tBu**. Naphthalene–acridines are classic TADF materials with well-established solution TADF behavior and large intrinsic delayed fluorescence contribution under direct excitation,^37^ which enables direct comparison between conventional and sensitized excitation pathways.

Under direct photoexcitation in toluene, **NAI-SAF-4tBu** exhibits nanosecond prompt fluorescence followed by a delayed component (τ = 77 μs) that accounts for 91% of the total PL intensity (Figure 2 b). This behavior is consistent with efficient ISC and RISC under optical excitation. Full photophysical characterization is provided in the Supporting Information (Section S3).

**Figure 2:**
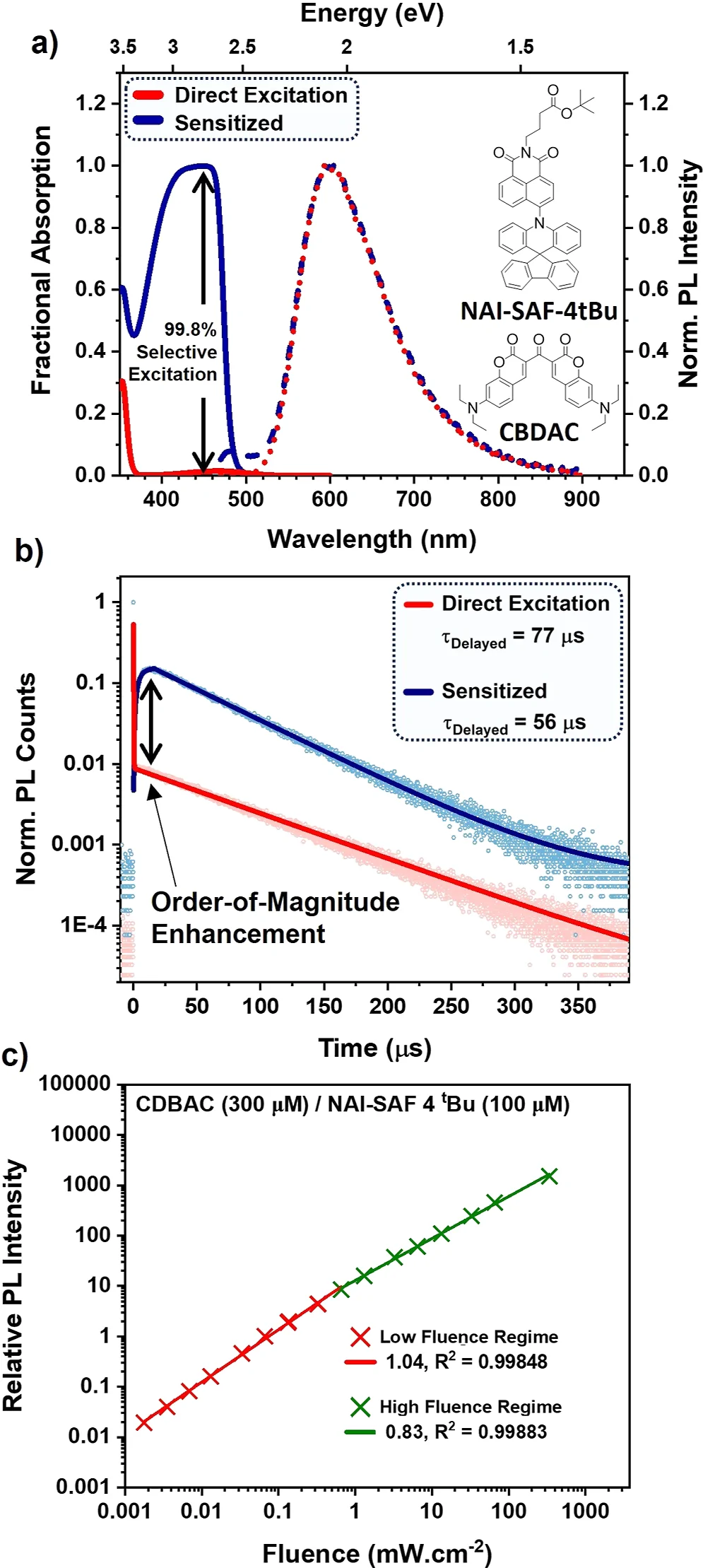
(A) Fractional absorption and normalized emission spectra for the toluene solutions studied via direct excitation and triplet sensitization; (b) Photoluminescence decays for direct excitation and sensitized samples; (c) Excitation fluence dependence for the sensitization experiment.

To decouple triplet population from intrinsic ISC, **NAI-SAF-4tBu** was studied with the organic triplet sensitizer **CBDAC**.^38^ **CBDAC** combines strong visible absorption (ε ca. 100,000 M^–1^ cm^–1^ at 450 nm), a high ISC yield (Φ_ISC_ = 0.92),^39^ an appropriate triplet energy (2.2 eV in toluene),^40^ and a nonphosphorescent triplet state at room temperature. The latter is critical: given the narrow singlet–triplet energy gap (ΔE_ST_) of TADF materials and the exothermic regime required for efficient triplet energy transfer (TET), phosphorescent sensitizers would be susceptible to Förster resonance energy transfer to the TADF analyte singlet state over distances exceeding those of molecular collisions, leading to unwanted singlet-state population and preventing selective population of the analyte triplet manifold.^41,42^

Excitation of a **CBDAC** (300 μM)/**NAI-SAF-4tBu** (100 μM) solution at 450 nm ensured near-exclusive sensitizer excitation (∼99.8% selectivity). As direct excitation of the TADF analyte is almost entirely suppressed, sensitizer-mediated triplet transfer is likely to become the dominant pathway by which the TADF molecule is excited. Consequently, we expected sensitization to strongly enhance triplet-state population within the TADF molecule without requiring quantitative triplet transfer, since TET need only outcompete the heavily suppressed direct excitation pathway rather than approach unity. Under sensitized excitation, the PL remains dominated by the TADF analyte (Figure 2 a). Time-resolved multichannel scaling (MCS) PL measurements reveal a delayed emission component with a lifetime comparable to that observed under direct excitation considering experimental error (τ = 56 μs), suggestive of delayed fluorescence arising from the same underlying RISC process as under direct excitation (Figure 2 b). Delayed emission accounts for 98.7% of the total emission intensity under sensitized excitation, corresponding to an order-of-magnitude suppression of prompt fluorescence, highlighting the effectiveness of selectively populating the triplet manifold to promote TADF.

In contrast to the characteristic prompt PL ‘spike’ observed under direct excitation, the sensitized system exhibits negligible prompt emission and a clear rise in the delayed PL signal before it decays (Figure 2 b). The rise time shortens systematically with increasing TADF analyte concentration, consistent with a collisional TET process and with a kinetic model in which the **NAI-SAF-4tBu** triplet state is initially populated via triplet transfer from **CBDAC** under sensitized excitation (Figure S4 and Section S10).

As TET from triplet sensitizers is ubiquitous in triplet–triplet annihilation (TTA) systems,^38,40^ it is essential to distinguish the present mechanism from TTA. In the low-fluence regime relevant to all steady-state and time-resolved measurements, the delayed PL intensity exhibits a linear dependence on excitation fluence (slope ca. 1; Figure 2 c), consistent with the proposed single-triplet TADF mechanism.^43^ TTA would require a quadratic dependence.^44^ Deviations from linearity are observed only at higher fluences, indicating the onset of additional nonradiative pathways; these regimes were avoided in all subsequent experiments.

Together, these data establish that diffusional TET from an organic sensitizer can provide direct optical access to the triplet manifold of a TADF emitter, yielding delayed fluorescence that is mechanistically distinct from TTA and without perturbing the intrinsic RISC process. Similar results were obtained for **NAI-SAF-4tBu** using the higher-triplet-energy (>2.7 eV) sensitizer **Michler’s Ketone** under UV excitation (375 nm), demonstrating that the approach is versatile across different sensitizers and excitation wavelengths (Figure S7).

Having established the validity of triplet sensitization, we next apply the method to a set of contemporary high radiative rate (*k*_r_^S^ ca. 10^8^ s^–1^) TADF emitters to evaluate generality across diverse molecular structures, emission colors from blue to near-infrared, and delayed-emission lifetimes spanning a wide temporal range. Sample preparation considerations and details are provided in the Supporting Information Sections S1 and S3.

We consider two widely studied, yet structurally and photophysically distinct, TADF emitters: the blue multiresonance (MR) emitter ν**-DABNA** and the NIR D–A–D emitter **BF2** (Figure 3). ν**-DABNA** represents an archetype for high-efficiency blue MR-TADF–at the time of writing (April. 2025), the original ν**-DABNA** study by Hatakeyama et al. had been cited >1400 times.^45^ ν**-DABNA** combines fast radiative decay with a small ΔE_ST_, yielding measurable delayed fluorescence in solution under direct excitation, albeit with a relatively short delayed lifetime on the order of a few microseconds. In contrast, **BF2** is a high-performance NIR TADF emitter whose exceptionally large radiative rate strongly suppresses intrinsic ISC, resulting in only a minute, but long-lived, delayed fluorescence contribution in toluene solution (780 μs, ca. 0.1% contribution) that lies close to the limits of experimental detectability (Figure S6). To our knowledge, delayed fluorescence from **BF2** in apolar solvent has not been detected prior to this work, reflecting the severe suppression of intrinsic ISC in this system.^14,46,47^

**Figure 3:**
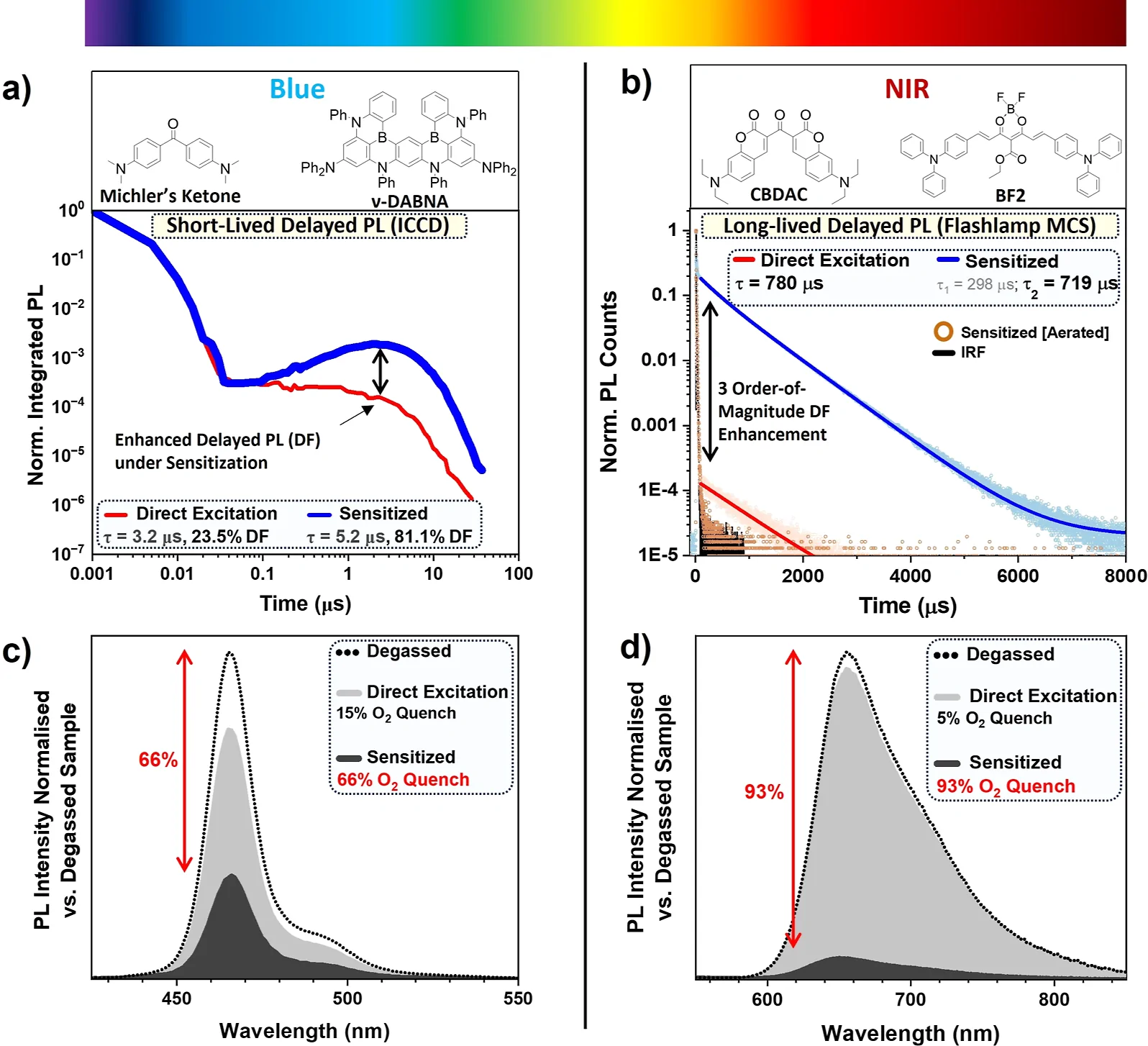
(A) Time-resolved PL decay for sensitized Michler’s Ketone (600 μM)/ν-DABNA (40 μM); (b) Time-resolved PL decay for sensitized CBDAC (300 μM)/BF_2_ (100 μM); (c) Oxygen dependence of PL quenching for Michler’s Ketone (600 μM)/ν-DABNA (40 μM); (d) Oxygen dependence of PL quenching for CBDAC (300 μM)/BF_2_ (100 μM). All solutions were prepared in toluene.

Despite their photophysical differences, triplet sensitization enhances access to delayed fluorescence in both systems, indicated via both time-resolved PL and steady-state PL oxygen quenching (Figure 3).

For ν**-DABNA**, sensitized excitation using **Michler’s ketone** (λ_exc_ = 350 nm) substantially increases the delayed fluorescence contribution measured with pulsed laser excitation and an ICCD camera (24% → 81% contribution), while preserving a delayed lifetime comparable to that observed under direct excitation in our hands and the literature (Figure 3 a).^45,48^ We note that the delayed PL rise associated with the sensitization experiment enables the delayed PL of ν**-DABNA** to also be measured with a microsecond flashlamp setup despite its short delayed lifetime (Figure S8).

For **BF2**, sensitization (**CBDAC**, λ_exc_ = 450 nm) produces a dramatic enhancement in delayed fluorescence detectability (Figures 3 b and S2). The delayed emission in the sensitized sample has an intensity of ∼10^–1^ relative to the intensity of the peak prompt emission. This corresponds to a 3 orders of magnitude enhancement versus the relative intensity (∼10^–4^) of the delayed emission to the prompt signal under direct excitation, converting a challenging experiment at the limits of instrumental sensitivity into a routine measurement. MCS reveals a biexponential decay comprising a dominant long-lived component (719 μs) that closely matches the delayed lifetime observed under direct excitation. A shorter component (298 μs) is observed as an experimental artifact, which arises from convolution of the instrument response (IRF) with the rise kinetics associated with TET and vestigial prompt emission–an assignment supported by kinetic modeling (Section S10). The preservation of the long-lived component, together with the linear excitation-fluence dependence of the delayed emission (Figure S13), confirms that sensitization enhances sensitivity without perturbing the intrinsic RISC process.

The intrinsic delayed fluorescence lifetime observed here (>700 μs) is substantially longer than that previously reported for **BF2** in doped films, providing key support to previous claims that solid state effects such as aggregation and spontaneous exciton dissociation drive RISC in device-relevant films.^14,46^

In summary, analysis of ν**-DABNA** and **BF2** demonstrates that triplet sensitization provides a route for enhanced access to the triplet photophysics of TADF compounds that exhibit delayed fluorescence across disparate experimental regimes: covering the visible spectrum with delayed-lifetime regimes spanning over 2 orders of magnitude, independent of the specific time-resolved detection technique employed. Additionally, our first observation of toluene solution TADF for **BF2**—made experimentally trivial through triplet sensitization—provides a new validation for the importance of intermolecular TADF mechanisms for **BF2** in the solid state.

We next apply the approach to extend photophysical characterization beyond the limits imposed by conventional direct excitation. We focus on **dpTPAAP**,^49^ a representative member of the rigid π-extended pyrazine acceptor family–materials that have consistently defined the state of the art for red-to-near-infrared TADF OLED performance (Figure 4).^28,36,49–81^

**Figure 4:**
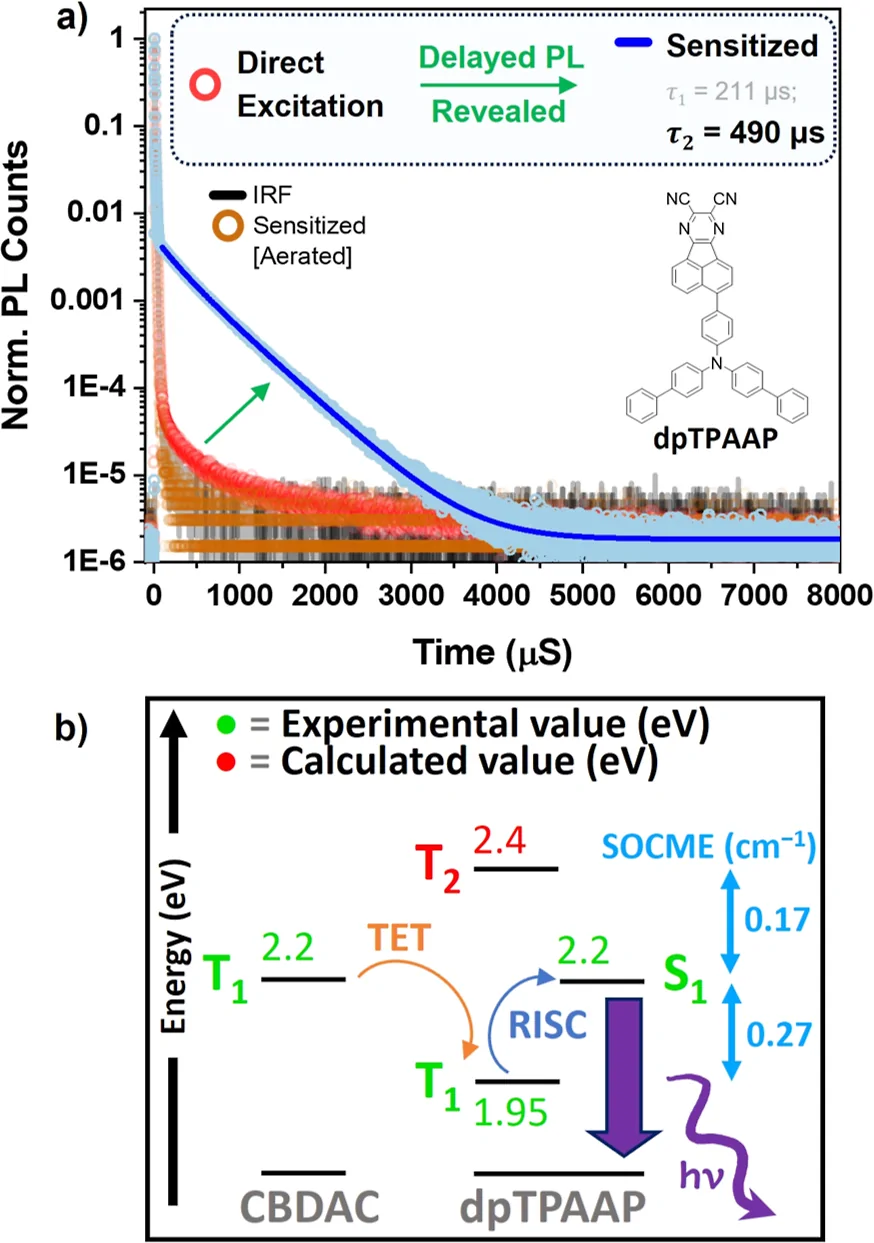
(A) Photoluminescence decays to highlight that delayed fluorescence in toluene solution is revealed for dpTPAAP via triplet sensitization; (b) Jablonski diagram for the sensitization of dpTPAAP in solution constructed from experimental and computational data.

In toluene solution, **dpTPAAP** exhibits a narrow singlet–triplet energy gap (ΔE_ST_ = 0.25 eV)^49^ and an exceptionally high PL quantum yield (Φ_PL_ = 99%) with a large radiative rate (*k*_r_^S^ = 1.1 × 10^8^ s^–1^). While efficient TADF has been reported for **dpTPAAP** in thin films,^49^ we were unable to detect appreciable delayed fluorescence in toluene under direct excitation using a sensitive ICCD detector. Moreover, when examined using microsecond flashlamp-excited MCS, the PL decay was near-indistinguishable from the instrument response function (Figure 4 a). The absence of analyzable solution-state delayed fluorescence for **dpTPAAP** under direct excitation is consistent with literature,^49^ and more broadly reflects a recurring observation for π-extended pyrazine-based NIR TADF emitters, for which solution-state delayed fluorescence is frequently not reported^51–53,59,67,68,71,77,79,80^ or explicitly stated to be unobservable.^56,58,62,65,72,73,82,83^

The photophysics of donor–acceptor TADF molecules are well established to be sensitive to their local environment,^43,84^ and the delayed fluorescence of many π-extended pyrazine-based NIR emitters has been linked to solid-state effects such as J-aggregation or intermolecular charge transfer interactions.^49,65,72,73,82^ In this context, the lack of observable delayed fluorescence in solution could be taken to imply that any intrinsic triplet harvesting capability is absent, with mechanistic attention instead focused directly on solid-state behavior and device performance. However, an alternative—and largely untested—possibility is that RISC pathways are intrinsically present in these molecules but rendered optically inaccessible under direct excitation in solution due to Φ_ISC_ ≈ 0.

Triplet sensitization allows this ambiguity to be resolved. Upon sensitized excitation with **CBDAC** (λ_exc_ = 450 nm), delayed fluorescence from **dpTPAAP** becomes easily observable using flashlamp-excited MCS (Figure 4 a). The delayed emission is fully quenched by oxygen and demonstrates a linear fluence dependence (Figure S14), confirming its TADF origin. A well-defined long-lived PL component with a lifetime of approximately 490 μs is observed.

To determine the RISC mechanism accessed in the sensitization experiment, we complemented the experimental results with computations (see Supporting Information, Section S9, for full computational details). The key energetic relationships are summarized schematically in the Jablonski diagram shown in (Figure 4 b). Considering the experimentally determined triplet energies of **CBDAC** ^40^ and **dpTPAAP** ^49^ in solution and the T_1_–T_2_ energy gap (≈0.45 eV) predicted by TD-DFT, exothermic TET to the **dpTPAAP** T_1_ is proposed to be more likely than energy transfer into the higher-energy triplet manifold.

The calculations also provide insight into the relative efficiencies of possible RISC pathways in **dpTPAAP**. The spin–orbit coupling matrix element (SOCME) calculated for the S_1_–T_1_ transition (0.27 cm^–1^) is larger than for S_1_–T_2_ (0.17 cm^–1^), indicating that direct T_1_ → S_1_ RISC is the more strongly coupled pathway. Taken together, energetics and the relative SOC strengths suggest that the delayed PL observed under sensitized excitation for **dpTPAAP** is likely to predominantly arise from intrinsic T_1_ → S_1_ RISC, rather than from the sensitizer-induced population of higher-lying triplet states previously reported by Gilch et al. for thioxanthone and acridone molecules.^85,86^

Taking the intrinsic delayed fluorescence lifetime (490 μs) and reasonably concluding that Φ_ISC_ ≈ 0 as Φ_PL_ = 99% with no delayed PL under direct excitation, an upper limit for the intrinsic *k*_RISC_ can be estimated as ≈2 × 10^3^ s^–1^ under the commonly employed assumption that *k*_RISC_ ≫ *k*_r_^T^ + *k*_*n*r_^T^.^15–17,87^ While this assumption should become less strictly valid at lower triplet energies or for smaller values of *k*_RISC_, it provides a reasonable approximation in the present case. Within the limitations of this assumption, comparison with **BF2** suggests that **dpTPAAP** may be an intrinsically more efficient triplet harvester, despite its apparent lack of solution-state TADF under direct excitation. **dpTPAAP** exhibits a shorter intrinsic delayed lifetime (490 vs 719 μs), a larger extracted *k*_RISC_ (2 × 10^3^ vs 1.3 × 10^3^ s^–1^), and a higher PL quantum yield (99 vs 90%) than **BF2**. Yet these properties could not be inferred from conventional direct excitation measurements, underscoring the potential of sensitization as a key diagnostic tool for modern high-radiative-rate TADF emitters.

Additionally, the intrinsic delayed fluorescence lifetime extracted here for **dpTPAAP** is comparable in magnitude to the longest delayed component previously reported in doped solid-state films (30 wt % in TPBi, τ = 380 μs), where highly multiexponential emission dynamics were observed.^49^ This correspondence enables assignment of the intrinsic molecular RISC time scale within otherwise complex solid-state behavior and access to a new direct link between solution-phase photophysics and device-relevant observations.

## Conclusion and Outlook

The characterization of thermally activated delayed fluorescence (TADF) relies fundamentally on the population and interrogation of triplet excited states. Under direct optical excitation, triplet formation is governed by the intrinsic intersystem crossing yield (Φ_ISC_) of the emitter. As TADF materials continue to expand into regimes of increasingly low intrinsic Φ_ISC_, for example through the development of high-radiative-rate emitters, delayed fluorescence becomes progressively more difficult to detect using conventional photophysical approaches, even when reverse intersystem crossing (RISC) pathways may be present.

In this work, we have implemented a triplet energy transfer (TET) sensitization strategy that decouples triplet population from intrinsic ISC. By selectively populating the triplet manifold of TADF chromophores via diffusional TET from an organic sensitizer, any reliance on Φ_ISC_ is completely bypassed, enabling delayed fluorescence to be selectively promoted. Across a set of TADF emitters selected for their diversity in structure, emission color and delayed photoluminescence (PL) time scale, accessing the triplet manifold through sensitization enhances the detectability of delayed PL by up to 3 orders of magnitude relative to direct excitation, converting challenging experiments at the limits of instrument sensitivity into routine measurements. This enabled the facile determination of the intrinsic toluene solution delayed fluorescence lifetime and *k*_RISC_ for **BF2** for the first time, lending strong experimental support to prior reports that intermolecular RISC plays a dominant role in this system in the solid state.

The utility of the triplet sensitization approach is most clearly illustrated by **dpTPAAP**, a high-radiative-rate emitter belonging to a state-of-the-art family of near-infrared TADF materials for which solution-state delayed fluorescence has been commonly reported as unobservable. Through triplet sensitization, previously hidden delayed fluorescence could be detected in solution, enabling determination of the intrinsic delayed fluorescence lifetime and estimation of the intrinsic *k*_RISC_ parameter in a regime beyond the reach of conventional direct-excitation methods. The delayed fluorescence lifetime obtained in solution lies within the same temporal regime as the longest delayed component previously reported in doped solid-state films, providing a direct link between intrinsic solution-state photophysics and complex solid-state behavior by enabling deconvolution of the intrinsic molecular RISC time scale from multicomponent solid-state emission dynamics. More broadly, these results demonstrate that intrinsic TADF properties can remain highly relevant even within high-performing material families where delayed fluorescence has historically been only observed—or only reported—in the solid state.

The present work therefore demonstrates the broader value of experimental methodologies that provide direct access to intrinsic triplet-state photophysics. As the scope of TADF continues to expand to include materials with increasingly low intrinsic ISC yields, experimental approaches that decouple triplet population from ISC will become increasingly important for directly interrogating intrinsic triplet-state photophysics and advancing mechanistic understanding across an increasingly diverse range of TADF systems.

## Supplementary Material



## Notes

Synthetic details and additional experimental and computational data are included in the Supporting Information (PDF)
