# Supplementary material for: Decoupling Triplet Population from Intersystem Crossing in TADF Materials
Source: J Am Chem Soc. 2026 Sep 8;148(37):39801–10. doi: 10.1021/jacs.6c10012 (PMC13614300; doi:10.1021/jacs.6c10012)
Supplement: Supplementary file 1 [file ja6c10012_si_001.pdf]

Supporting Information

Decoupling Triplet Population from Intersystem Crossing in TADF Materials

Oliver Millington[‡,a,b], Erin M. Holdsworth[‡,a], Victor Gray[c], Tarig B. E. Mustafa[a,b], Sebastian Gorgon[b], Lars van Turnhout[b], Weixuan Zeng[a,d], Alexander J. Gillett[e], Akshay Rao[b], Daniel G. Congrave[f] and Hugo Bronstein[a,b]

[‡] Equal Contribution
[a] Department of Chemistry, University of Cambridge, Cambridge, CB2 1EW, UK.
[b] Cavendish Laboratory, University of Cambridge, Cambridge, CB3 0HE, UK.
[c] Department of Chemistry, Ångström Laboratory, Uppsala University, SE-751 20 Uppsala, Sweden.
[d] Zhangjiang Laboratory, Shanghai 201210, China.
[e] Department of Physics, Chemistry and Biology (IFM), Linköping University, 581 83 Linköping, Sweden.
[f] Chemistry Research Laboratory, University of Oxford, Oxford, OX1 3TA, UK.

Table of Contents

1. EXPERIMENTAL AND SYNTHETIC DETAILS 2

2. ABSORPTION SPECTRA: EXTINCTION COEFFICIENTS 7

3. PHOTOPHYSICAL PARAMETERS FOR NAI-SAF 4 ^{t}Bu 11

4. PHOTOPHYSICAL PARAMETERS FOR BF2 11

5. PHOTOPHYSICAL PARAMETERS FOR DPTPAAP 12

6. ADDITIONAL TIME-RESOLVED PHOTOLUMINESCENCE DATA 17

7. ADDITIONAL OXYGEN QUENCHING DATA 21

8. ADDITIONAL EXCITATION FLUENCE DATA 22

9. COMPUTATIONAL CHEMISTRY 24

10. KINETIC MODELLING 27

11. REFERENCES 32

S1. Experimental and Synthetic Details

General

All photophysical characterisation measurements in this work were carried out on solutions in toluene.

Absorption spectroscopy was measured using either a Shimadzu UV1800 or UV3600 spectrometer. Steady-state PL spectra were obtained using an Edinburgh instruments FS5. TCSPC data were obtained on an Edinburgh instrument FS5 or FLS1000 spectrometer, while MCS data were obtained exclusively on an Edinburgh instruments LFS spectrometer. Excitation sources for TCSPC and MCS were either a 450 nm pulsed diode laser or a monochromated microsecond flashlamp. Photoluminescence quantum yields were determined on an Edinburgh instruments FS5 in an integrating sphere,[1,2] with correction for self-absorption losses.[3] Additional TRPL and fluence-dependence data were recorded using an Andor electrically gated intensified charge-coupled device (ICCD) as the detector. The samples were excited off-axis using appropriately filtered wavelength tuneable pump pulses obtained from Orpheus-Lyra (Light Conversion) driven by Pharos (Light Conversion) set to a repetition rate of 1 kHz. The emission was collected, filtered and injected into a calibrated grating spectrometer (Andor SR303i) and detected using an electrically-gated intensified CCD camera (Andor iStar DH740 CCI-010). Overlapping time regions were used to compose the decays at multiple constant gate widths.

^{1}H NMR spectra were recorded on a 400 MHz Avance III HD Spectrometer or 400 MHz Smart Probe Spectrometer in the stated solvent using residual protic solvent as the internal standard. ^{1}H NMR chemical shifts are reported to the nearest 0.1 ppm. The coupling constants (J) are measured in Hertz. ^{13}C NMR spectra were recorded on the 500 MHz DCH Cryoprobe Spectrometer in the stated solvent using the residual protic solvent as the internal standard. ^{13}C NMR chemical shifts are reported to the nearest 0.01 ppm. Mass spectra were obtained using a Waters LCT, Finnigan MAT 900XP or Waters MALDI micro MX spectrometer at the Department of Chemistry, University of Cambridge. Thin layer chromatography (TLC) was carried out on silica gel and visualized using UV light (254, 365 nm). Flash chromatography was carried out on a Biotage Isolera automated flash chromatography machine on 60-micron silica gel cartridges purchased from Biotage. Microwave reactions were run in a Biotage Initiator reactor.

All commercial chemicals were of ≥95% purity and were used as received without further purification. Anhydrous solvents were purchased from Sigma Aldrich or Acros Organics and used as received. Michler's ketone (Sigma Aldrich), CDBAC (Tokyo Chemical Industry) and ν-DABNA (Lumtec) were obtained commercially.

BF_2 was prepared according to a literature procedure.[4] dpTPAAP was prepared according to a literature procedure.[5]

Oxygen exclusion

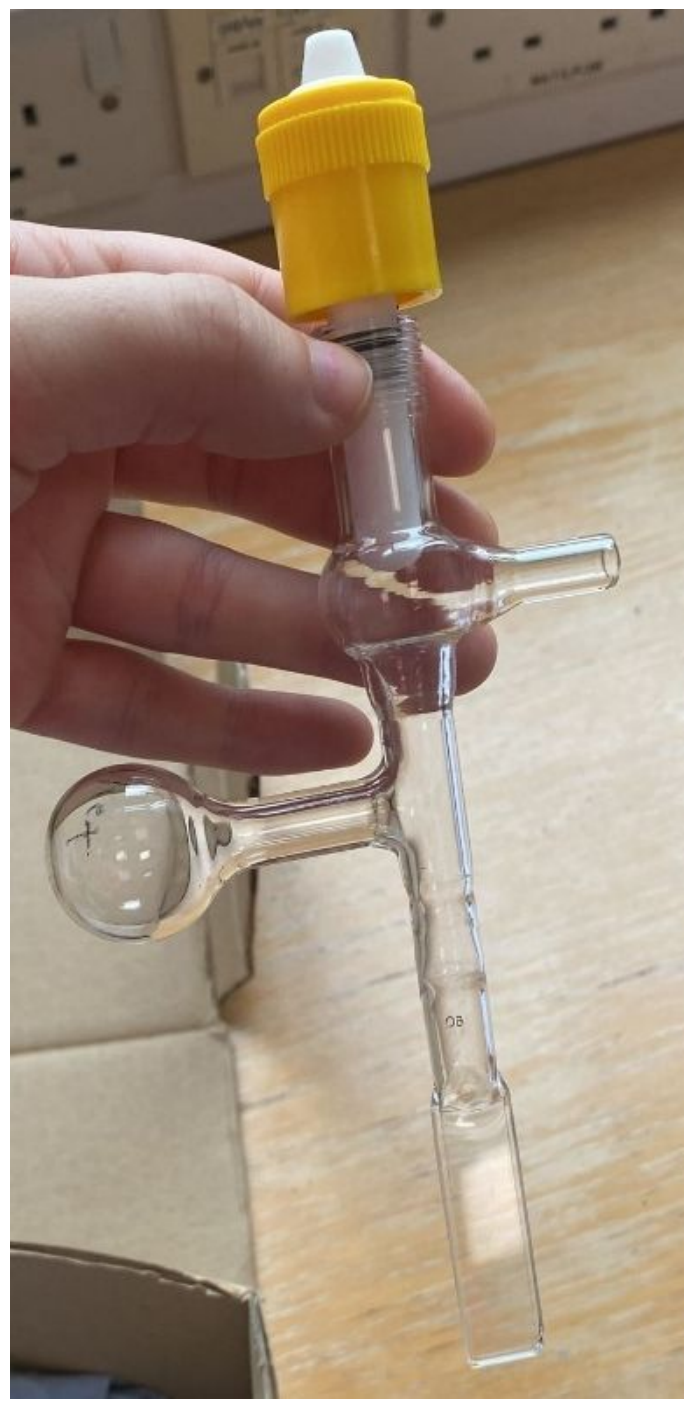

Exclusion of oxygen was paramount for this study due to the presence of long-lived triplet species.[6] To avoid excessively high optical densities and facilitate a broader range of molar analyte concentrations, the PL experiments were run in 1 mm pathlength quartz fluorescence cuvettes. Cuvettes with quartz to glass graded seals were purchased from Starna Scientific and fitted with a J. Youngs tap and degassing chamber by a glassblower. In-situ degassing of the sample solutions via repeated freeze pump thaw cycles using a high vacuum pump (10^{-3} mbar) is enabled with high reproducibility. *While we employed customized glassware here to ensure rigorous oxygen-free conditions, we note that exclusion of oxygen is a common necessity to any delayed fluorescence experiment. Hence, the main practical experimental difference between our method and conventional direct excitation is the preparation of a binary solution of sensitizer and analyte.*

Synthesis

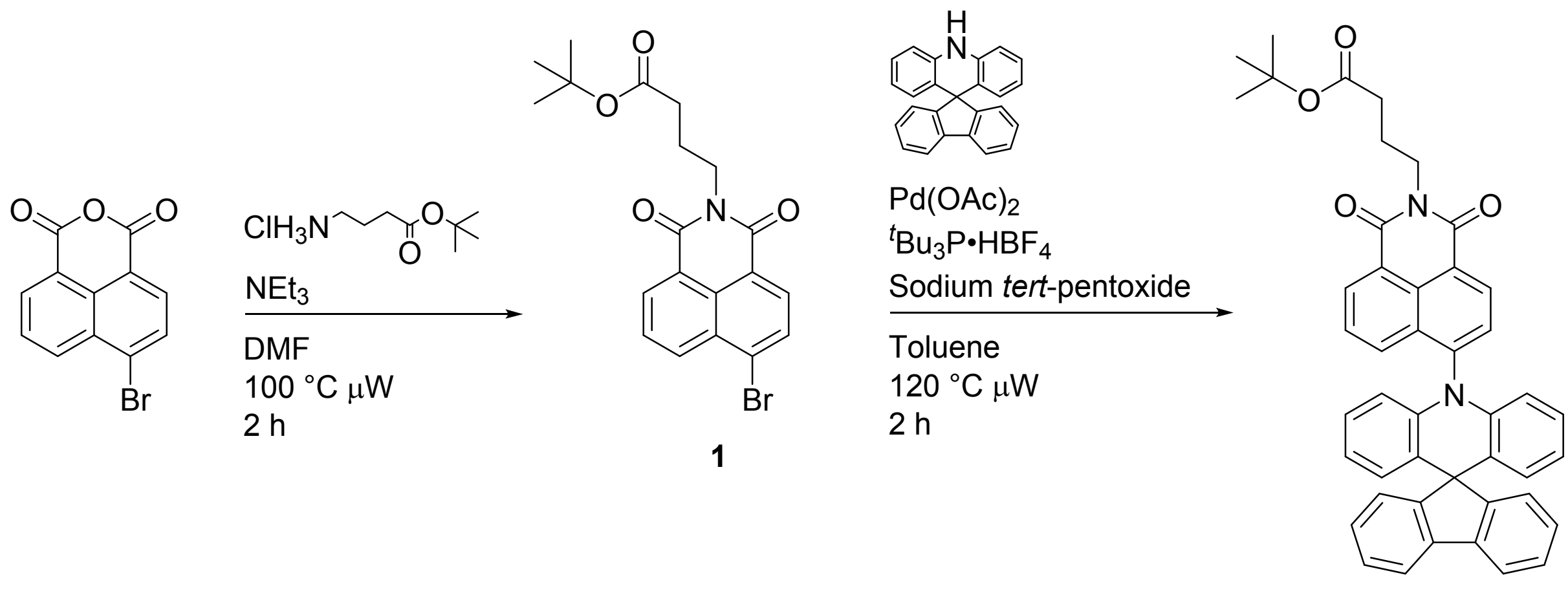

Scheme S1. Synthesis of NAI-SAF-4 ᵗBu.

1. 4-Bromo-1,8-naphthalic anhydride (550 mg, 2.00 mmol, 1.00 eq.), tert-butyl 4-aminobutanoate hydrochloride (470 mg, 2.40 mmol, 1.20 eq.) and triethylamine (0.35 mL) were combined in DMF (10 mL) in a crimp cap microwave vial which was sealed under argon. The mixture was heated at 100 °C

for 2 h in a microwave reactor, cooled to room temperature and poured into a mixture of diethyl ether (50 mL) and 1 M HCl (50 mL). The layers were separated and the aqueous layer extracted twice more with diethyl ether (2 × 20 mL). The organic extracts were combined, dried over $MgSO_4$, filtered and the solvent removed under reduced pressure. The residue was purified by flash chromatography on silica gel (eluent: gradient 0:1 → 9:1 DCM/ EtOAc v/v) and the material obtained as the major fraction was triturated with MeOH at −78 °C to obtain **1** as a light-yellow powder (600 mg, 1.43 mmol, 72%). ^{1}H NMR data were in agreement with those reported in the literature.[7] ^{1}H NMR (400 MHz, $CDCl_3$) δ (ppm) = 8.65 (d, *J* = 7.3 Hz, 1H), 8.57 (d, *J* = 8.5 Hz, 1H), 8.41 (d, *J* = 7.9 Hz, 1H), 8.04 (d, *J* = 7.9 Hz, 1H), 7.84 (t, *J* = 7.9 Hz, 1H), 4.22 (t, *J* = 7.3 Hz, 2H), 2.35 (t, *J* = 7.3 Hz, 2H), 2.03 (p, *J* = 7.3 Hz, 2H), 1.42 (s, 9H).

NAI-SAF-4 ^{t}Bu. 10H-Spiro[acridan-9,9'-fluorene] (300 mg, 0.90 mmol, 1.50 eq.), **1** (251 mg, 0.60 mmol, 1.00 eq.), $Pd(OAc)_2$ (8 mg, 6 mol.%), $^t Bu_3P$•HBF_4 (32 mg, 18 mol.%) and sodium *tert*-pentoxide (118 mg, 1.07 mmol, 1.78 eq.) were combined in an oven dried crimp cap microwave vial which was sealed under argon. Degassed dry toluene (5 mL) was added and the reaction mixture was heated at 120 °C for 2 h in a microwave reactor. The reaction mixture was cooled to room temperature and poured into a mixture of DCM (50 mL) and 1 M HCl (50 mL). The layers were separated and the aqueous layer extracted twice more with DCM (2 × 20 mL). The organic extracts were combined, dried over $MgSO_4$, filtered and the solvent removed under reduced pressure. The residue was purified by flash chromatography on silica gel (eluent: gradient 0:1 → 1:0 DCM/ *n*-hexane v/v then 1:9 EtOAc/ DCM v/v) The orange band was collected and the material obtained upon evaporation of the column solvent was suspended in ethanol (20 mL) and refluxed for 1 h. The suspension was filtered hot and the filtrand washed with further ethanol (3 x 10 mL) to afford **NAI-SAF-4 ^{t}Bu** as a yellow powder (222 mg, 0.33 mmol, 55%). ^{1}H NMR (400 MHz, $CDCl_3$) δ (ppm) = 8.91 (d, *J* = 7.6 Hz, 1H), 8.71 (d, *J* = 7.2 Hz, 1H), 8.20 (d, *J* = 8.4 Hz, 1H), 7.98 (d, *J* = 7.6 Hz, 1H), 7.88 (d, *J* = 7.5 Hz, 1H), 7.83 (d, *J* = 7.5 Hz, 1H), 7.76 (t, *J* = 7.8 Hz, 1H), 7.45 (dt, *J* = 22.3, 8.8 Hz, 3H), 7.37 (dt, *J* = 11.5, 5.7 Hz, 2H), 7.29 – 7.23 (m, 2H), 6.81 (t, *J* = 7.7 Hz, 2H), 6.59 (t, *J* = 7.5 Hz, 2H), 6.45 (d, *J* = 7.8 Hz, 2H), 6.03 (d, *J* = 8.3 Hz, 2H), 4.33 (t, *J* = 7.1 Hz, 2H), 2.42 (t, *J* = 7.4 Hz, 2H), 2.13 (p, *J* = 7.2 Hz, 2H), 1.46 (s, 8H); ^{13}C NMR (101 MHz, $CDCl_3$) δ (ppm) 172.2, 163.9, 163.6, 157.8, 156.6, 144.0, 140.6, 140.3, 138.3, 132.8, 132.1, 130.8, 130.5, 130.4, 130.1, 128.6, 128.5, 128.5, 128.2, 127.8, 127.7, 127.5, 126.2, 125.5, 124.5, 123.9, 123.2, 121.3, 120.0, 114.6, 80.4, 56.6, 39.8, 33.3, 28.1, 23.6; HRMS (ASAP): *m/z* 669.2737 [M–H^+]. Calcd. for $C_{45}H_{37}N_2O_4^+$: 669.2753.

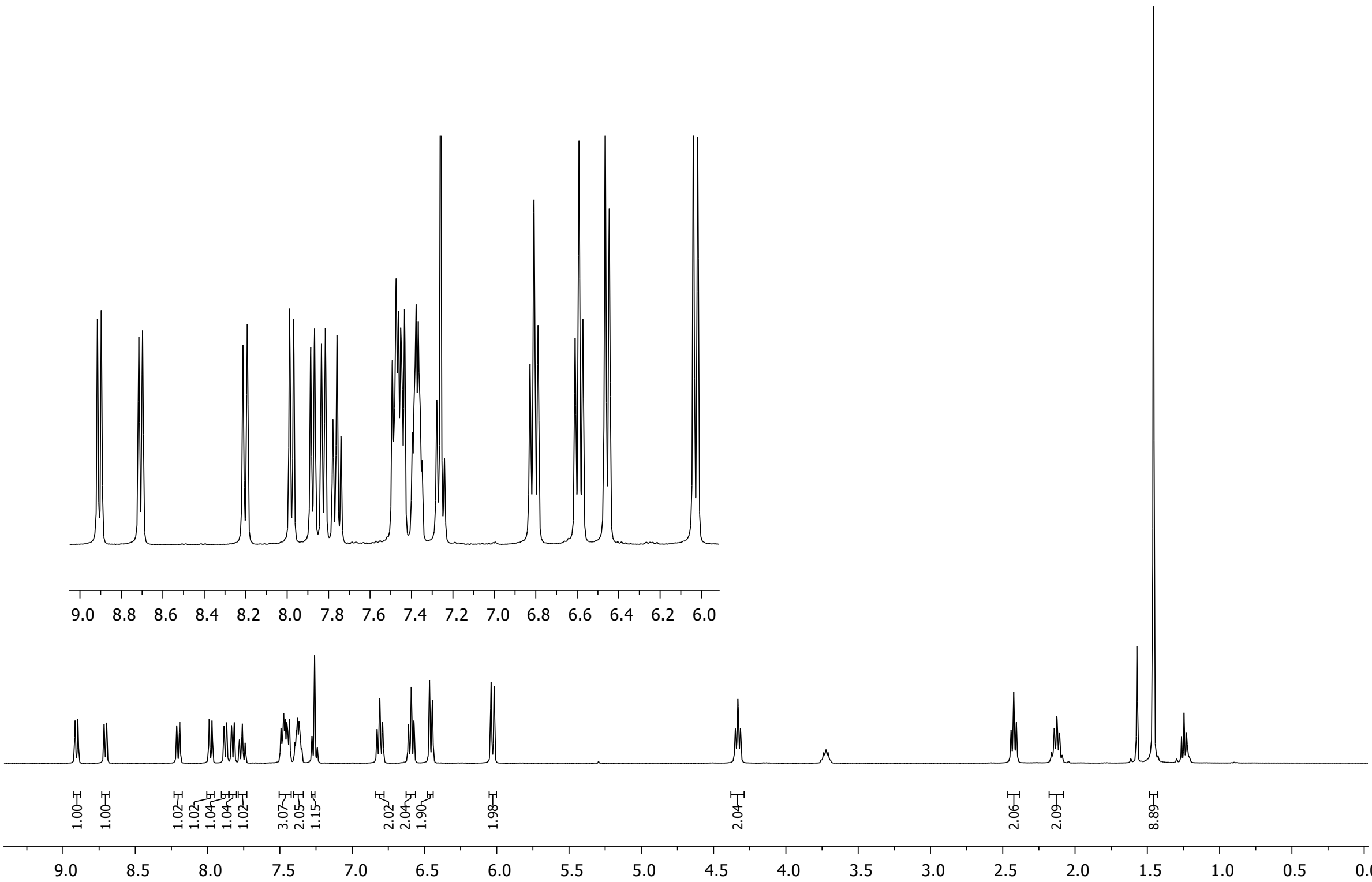
9.0 8.8 8.6 8.4 8.2 8.0 7.8 7.6 7.4 7.2 7.0 6.8 6.6 6.4 6.2 6.0
1.00
1.00
1.02
1.02
1.04
1.04
1.02
3.07
2.05
1.15
2.02
2.04
1.90
1.98
2.04
2.06
2.09
8.89
9.0 8.5 8.0 7.5 7.0 6.5 6.0 5.5 5.0 4.5 4.0 3.5 3.0 2.5 2.0 1.5 1.0 0.5 0.0

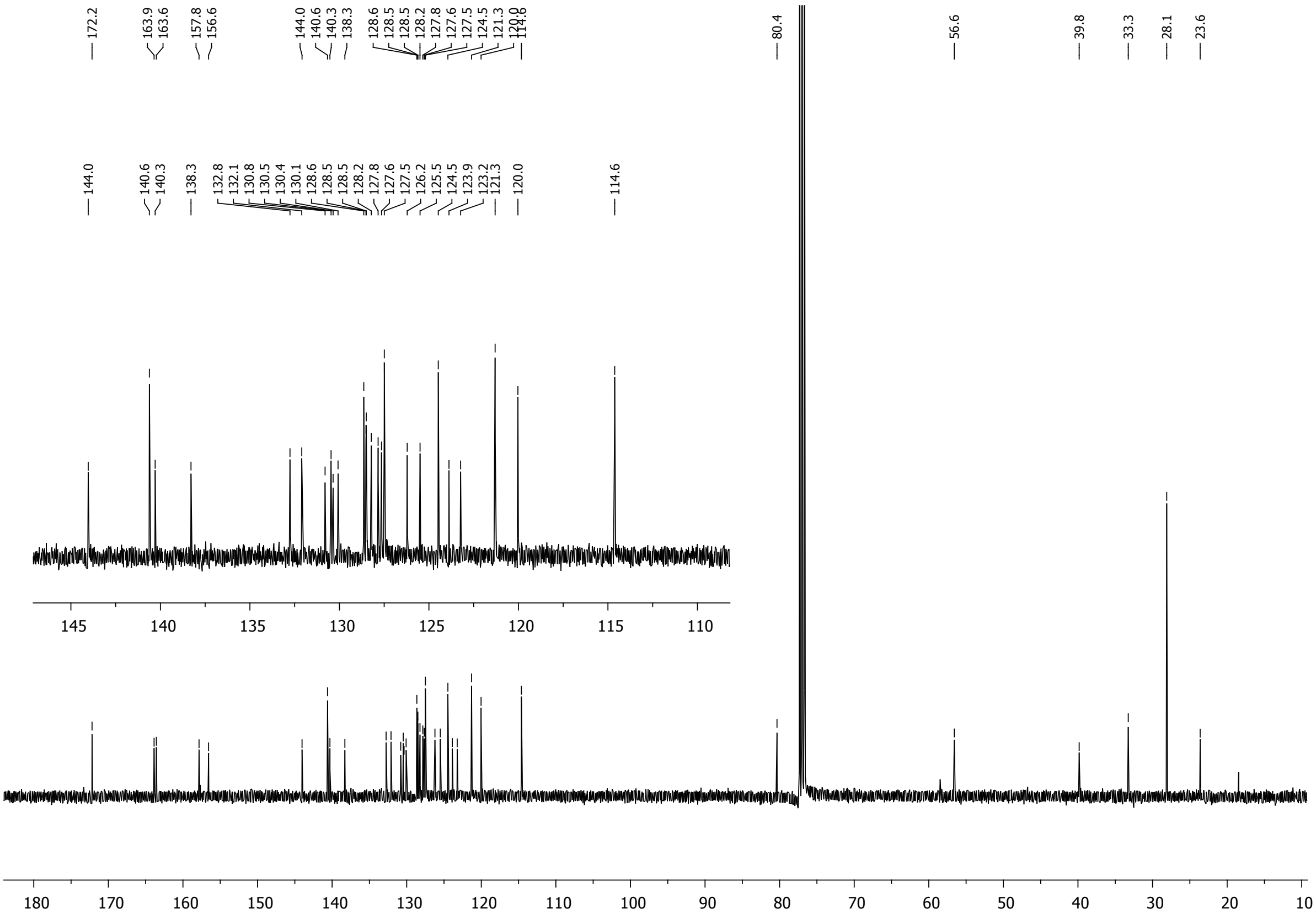
172.2
163.9
163.6
157.8
156.6
144.0
140.6
140.3
138.3
128.6
128.5
128.5
128.2
127.8
127.6
127.5
124.5
121.3
120.0
114.6
80.4
56.6
39.8
33.3
28.1
23.6
144.0
140.6
140.3
138.3
132.8
132.1
130.8
130.5
130.4
130.1
128.6
128.5
128.5
128.2
127.8
127.6
127.5
126.2
125.5
124.5
123.9
123.2
121.3
120.0
114.6
145 140 135 130 125 120 115 110
180 170 160 150 140 130 120 110 100 90 80 70 60 50 40 30 20 10

Elemental Composition Report

Single Mass Analysis

Tolerance = 500.0 PPM / DBE: min = -1.5, max = 50.0
Element prediction: Off
Number of isotope peaks used for i-FIT = 3

Monoisotopic Mass, Odd and Even Electron Ions
4 formula(e) evaluated with 1 results within limits (up to 50 best isotopic matches for each mass)
Elements Used:
C: 0-45 H: 0-37 N: 0-2 O: 0-4

HAB 46595 D CONGRAVE DGC-5-73 (LABELLED)
HAB_46595 D CONGRAVE DGC-5-73 (LABELLED) 1979 (4.260) Cm (1810:2007)
1: TOF MS ASAP+
4.28e+005

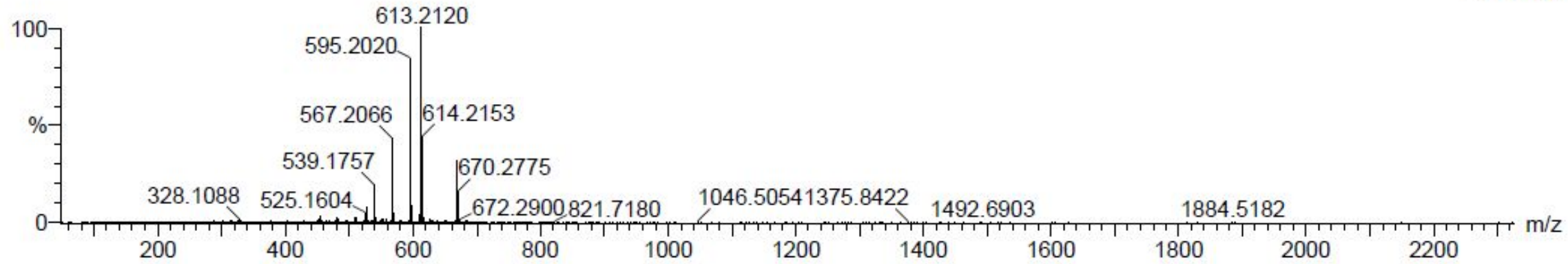

Minimum:				-1.5				
Maximum:		5.0	500.0	50.0				
Mass	Calc. Mass	mDa	PPM	DBE	i-FIT	Norm	Conf(%)	Formula
669.2737	669.2753	-1.6	-2.4	28.5	409.0	n/a	n/a	C45 H37 N2 O4

S2. Absorption Spectra: Extinction Coefficients

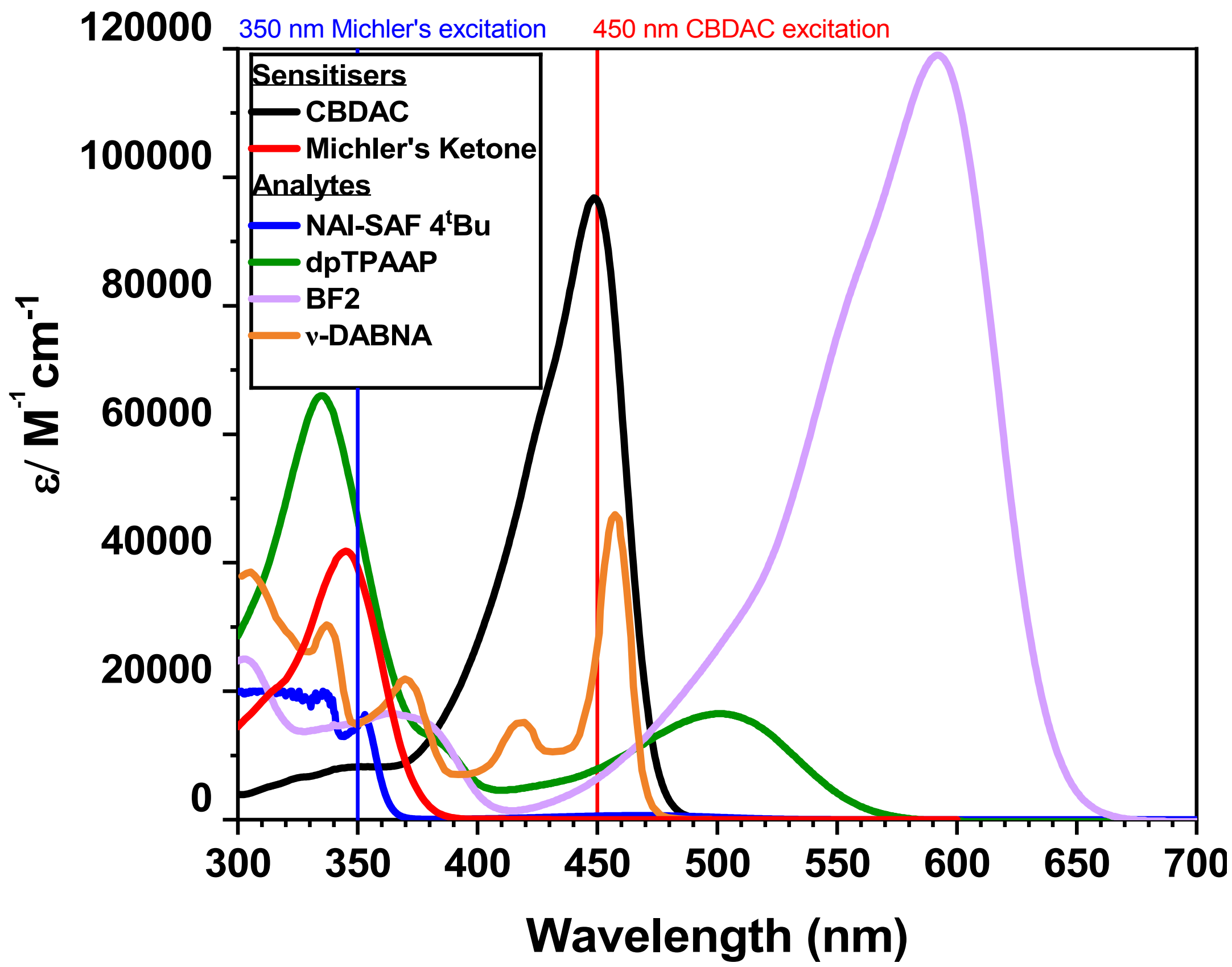

Figure S1. UV-Vis absorption spectra of toluene solutions of sensitisers and TADF analytes.

Table S1. Extinction coefficients at 450 nm for CBDAC-sensitised experiments, and the S_1 band of each material.

	$\lambda_{S1,peak}$/ nm	$\varepsilon_{S1,peak}$ / $M^{-1}.cm^{-1}$	ε_{450} / $M^{-1}.cm^{-1}$
**CBDAC**	449	96800[a]	**96400**
**NAI-SAF 4 ᵗBu**	467	640	**575**
**dpTPAAP**	500	16500[b]	**7900**
**BF2**	592	119000	**6400**

a. In good agreement with the literature value of 92,000 $M^{-1}.cm^{-1}$.[8]

b. In moderate agreement with the value indicated by the absorption data plotted by Xue *et al.*[5]

Table S2. Extinction coefficients for Michler's ketone-sensitised experiments, and the S_1 band of each material.

	**$\lambda_{S1,peak}$/ nm**	**$\varepsilon_{S1,peak}$ / $M^{-1}.cm^{-1}$**	**ε_{350} / $M^{-1}.cm^{-1}$**	**ε_{375} / $M^{-1}.cm^{-1}$**
**Michler's Ketone**	345	41800	**39200**	**4600**
**NAI-SAF 4 ^{t}Bu**	467	640	**-**	**95**
**ν-DABNA**	457	47500[a]	**14700**	**-**

a. Taken from work by Park and Yasuda *et al*.[9]

Excitation Selectivity for The Sensitizer in Mixed Solution Samples

Table S3. Excitation selectivity at 450 nm, for mixed solutions of CBDAC (300 μM) and NAI-SAF 4 ^{t}Bu of varying concentration.

[NAI-SAF 4 ^{t}Bu]/ μM	NAI-SAF 4 ^{t}Bu α @450 nm	CBDAC α[a] @450 nm	Percentage of light absorbed by CBDAC @ 450 nm/ %
100	0.00198	0.997	99.8
200	0.00396	0.995	99.6
500	0.00983	0.989	99.0
1000	0.0195	0.979	98.1
2000	0.0382	0.961	96.2

a. Fractional absorption (α)

Table S4. Excitation selectivity at 450 nm, for mixed solutions of CBDAC (300 μM) and TADF (100 μM) for each TADF compound.

Compound	α @450 nm (Sens. Mixture)	CBDAC α @450 nm (Sens. Mixture)	Percentage of light absorbed by CBDAC @ 450 nm/ %
**NAI-SAF 4 tBu**	0.00198	0.997	99.8
**dpTPAAP**	0.0266	0.972	97.3
**BF2**	0.0216	0.977	97.8

Table S5. Excitation selectivity at 375 nm, for a mixed solutions of Michler's Ketone (300 µM) and NAI-SAF 4 tBu (100 µM).

Compound	α @375 nm (Sens. Mixture)	Michler's α @375 nm (Sens. Mixture)	Percentage of light absorbed by Michler's @ 375 nm/ %
**NAI-SAF 4 tBu**	0.002	0.272	99.3

Table S6. Excitation selectivity at 350 nm, for a mixed solutions of Michler's Ketone (600 µM) and ν-DABNA (40 µM).

Compound	α @350 nm (Sens. Mixture)	Michler's α @350 nm (Sens. Mixture)	Percentage of light absorbed by Michler's @ 350 nm/ %
**ν-DABNA**	0.022	0.974	97.8

Methodology Example for Tables S3–S6

$\varepsilon_{CBDAC,450}$ / $M^{-1}.cm^{-1}$ = 96400

$\varepsilon_{NAI\text{-}SAF\ 4\ tBu,450}$ / $M^{-1}.cm^{-1}$ = 575

$A_{CBDAC,450} = 96400 \times (3 \times 10^{-4}) \times 0.1 = 2.893$

$A_{NAI\text{-}SAF\ 4\ tBu,450} = 570 \times (1 \times 10^{-4}) \times 0.1 = 0.00565$

$A_{Total,450} = 2.89865$

$\alpha_{Total,450} = 1 - 10^{-2.89865} = 0.9987$

$\alpha_{CBDAC,450} \sim \alpha_{Total,450} \times A_{CBDAC,450}/\ A_{Total,450} = 0.99675$

$\alpha_{NAI\text{-}SAF\ 4\ tBuC,450} \sim \alpha_{Total,450} \times A_{NAI\text{-}SAF\ 4\ tBu,450}/\ A_{Total,450} = 0.00198$

Percentage of light absorbed by sensitizer = $\alpha_{CBDAC,450}/\ \alpha_{Total,450} = 0.998$

S3. Photophysical Parameters for NAI-SAF 4 ᵗBu

Table S7. Experimental and derived photophysical parameters for NAI-SAF 4 ᵗBu in toluene under direct excitation

τ_P/ ns [a)]	10
τ_D/ µs [a)]	77
Φ_F / % [b)]	34
Prompt: Delayed PL Ratio [a)]	0.089:0.911
Φ_P / % [c)]	3.0
Φ_D / % [c)]	31
k_P/ s^{-1} [d)]	1.0×10^8
k_D/ s^{-1} [d)]	1.3×10^4
$k_{r,S}$ / s^{-1} [d)]	3.0×10^6
$k_{nr,S}$ / s^{-1} [d)]	5.9×10^6
k_{RISC} / s^{-1} [d)]	1.5×10^5
k_{ISC} / s^{-1} [d)]	9.1×10^7
Φ_{ISC} / % [d)]	91

a. Experimental parameters from TCSPC/ MCS experiments

b. Experimental parameter from PLQY measurement.

c. Directly derived from the experimental parameters.

d. Derived via applying an established methodology[10] for the determination of TADF parameters.

S4. Photophysical Parameters for BF2

Table S8. Experimental and derived photophysical parameters for BF2 in toluene under direct excitation

τ_P/ ns [a)]	2.4
τ_D/ µs [a)]	780
Φ_F / % [b)]	90
Prompt: Delayed PL Ratio [c)]	0.999:0.001
Φ_P / % [d)]	89.91
Φ_D / % [d)]	0.09
k_P/ s^{-1} [e)]	4.2×10^8
k_D/ s^{-1} [e)]	1.3×10^3
$k_{r,S}$ / s^{-1} [e)]	3.7×10^8
$k_{nr,S}$ / s^{-1} [e)]	4.2×10^7
k_{RISC} / s^{-1} [e)]	1.3×10^3
k_{ISC} / s^{-1} [e)]	4.2×10^5
Φ_{ISC} / % [e)]	0.1

a. Experimental parameters from TCSPC/ MCS experiments.

b. Experimental parameter from PLQY measurement.

c. Determined from integrated PL intensity measured on an ICCD camera.

d. Directly derived from the experimental parameters.

e. Derived via applying an established methodology[10] for the determination of TADF parameters.

S5. Photophysical Parameters for dpTPAAP

Table S9. Experimental and derived photophysical parameters for dpTPAAP in toluene

τ_P/ ns [a)]	8.9
τ_D/ μs [b)]	490
Φ_F / % [c)]	99
Prompt: Delayed PL Ratio [d)]	1:0
Φ_P / % [e)]	99
Φ_D / % [e)]	0
k_P/ s^{-1} [f)]	1.1×10^8
k_D/ s^{-1} [f)]	2.0×10^3
$k_{r,S}$ / s^{-1} [f)]	1.1×10^8
$k_{nr,S}$ / s^{-1} [f)]	1.2×10^6
k_{RISC} / s^{-1} [f)]	2.0×10^3
k_{ISC} / s^{-1} [f)]	0
Φ_{ISC} / % [f)]	0

a. Experimental parameter from TCSPC/ MCS experiments under direct excitation of dpTPAAP.
b. Experimental parameter from flash lamp MCS experiment on dpTPAAP-sensitized solution.
c. Experimental parameter from PLQY measurement under direct excitation of dpTPAAP.
d. No delayed PL observed on an ICCD camera. Delayed PL intensity ≈ 0.
e. Directly derived from the experimental parameters.
f. Derived via applying an established methodology[10] for the determination of TADF parameters.

S6. Additional Time-Resolved Photoluminescence Data

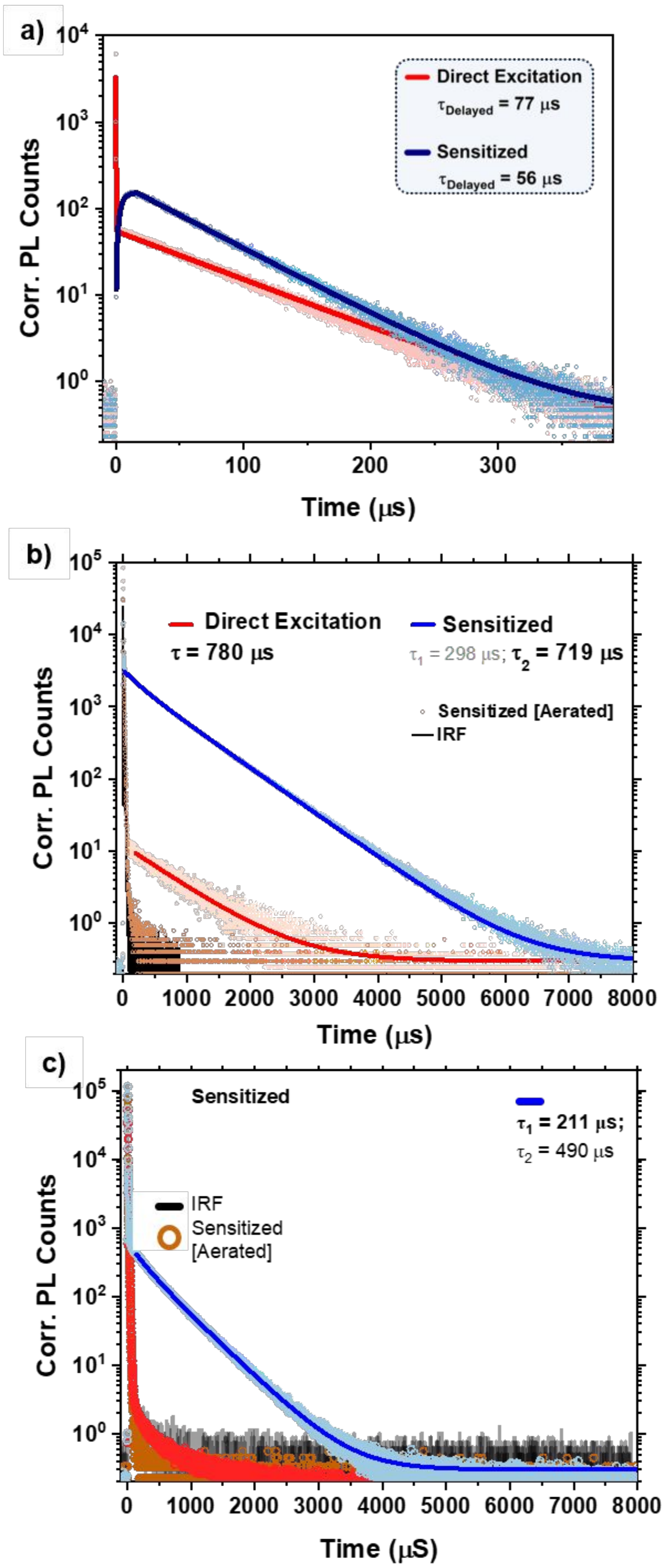

Figure S2. Time resolved PL traces normalised vs. peak dark counts at negative time. a) NAI-SAF 4 ^{t}Bu / CBDAC; b) BF2 w/ CBDAC; dpTPAAP w/ CBDAC.

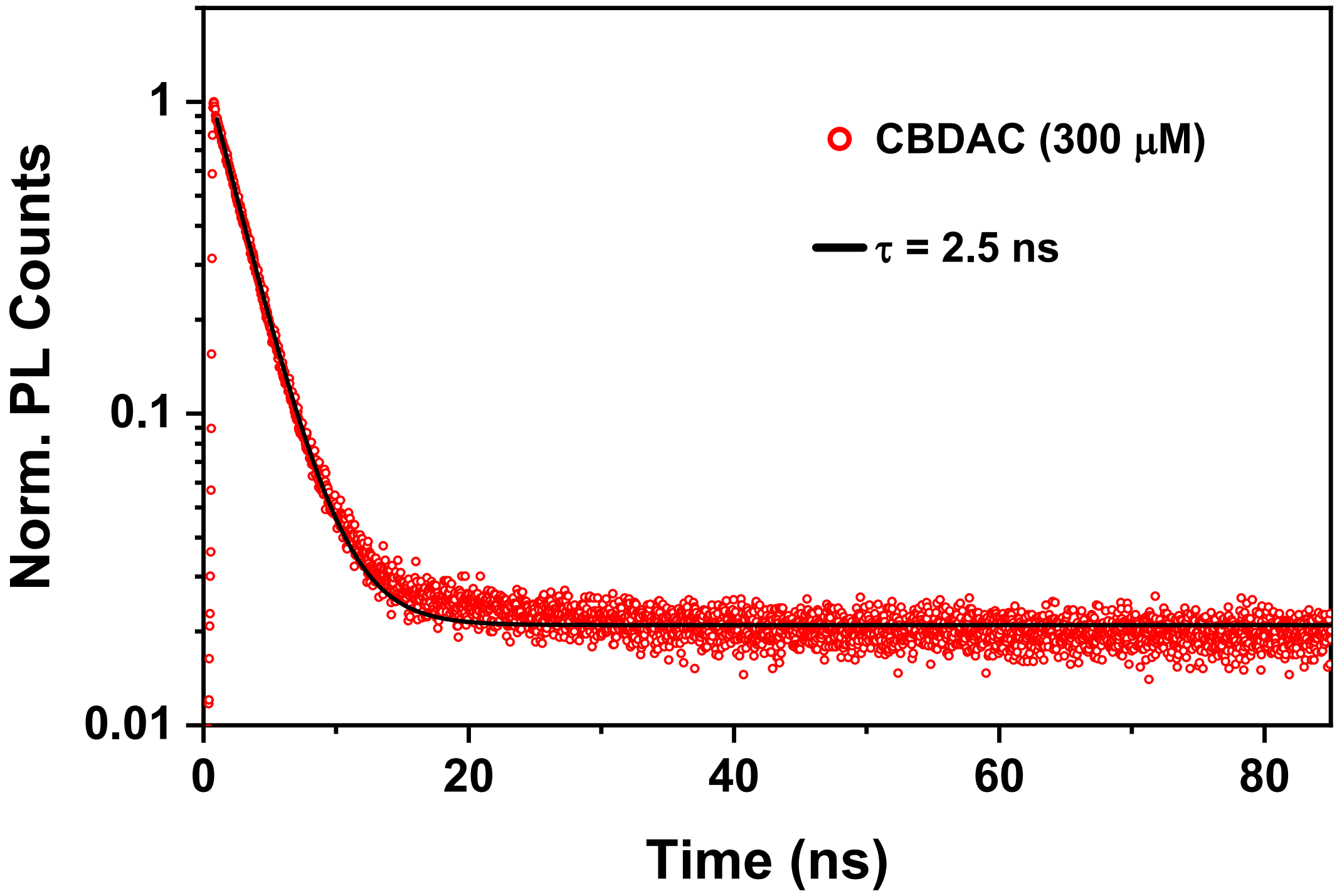

Figure S3. TCSPC trace for CBDAC in toluene solution excited at 450 nm.

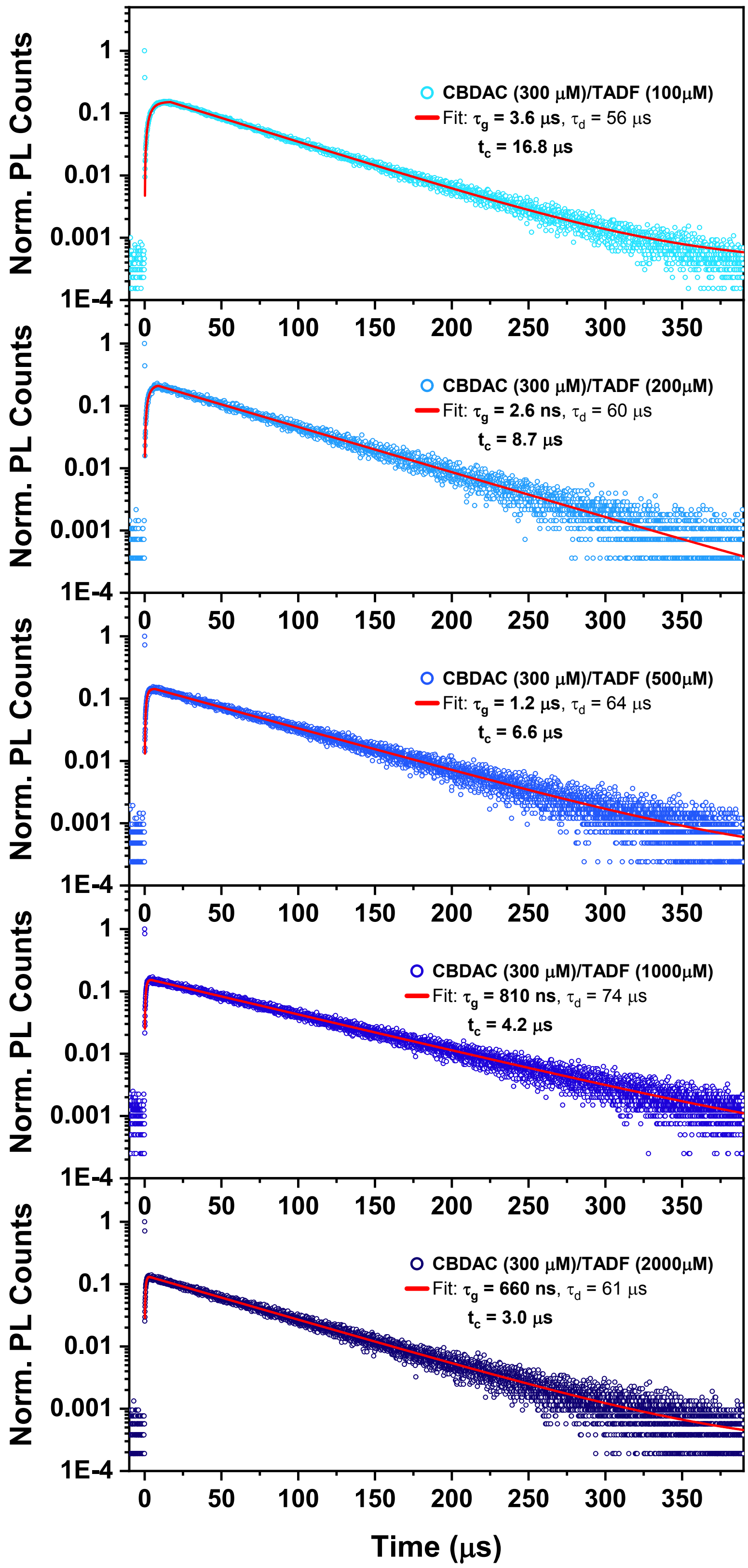

Figure S4. Full traces for PL decays at different concentrations of NAI-SAF 4 ᵗBu (CBDAC = 300 μM) measured using MCS and excited at 450 nm.

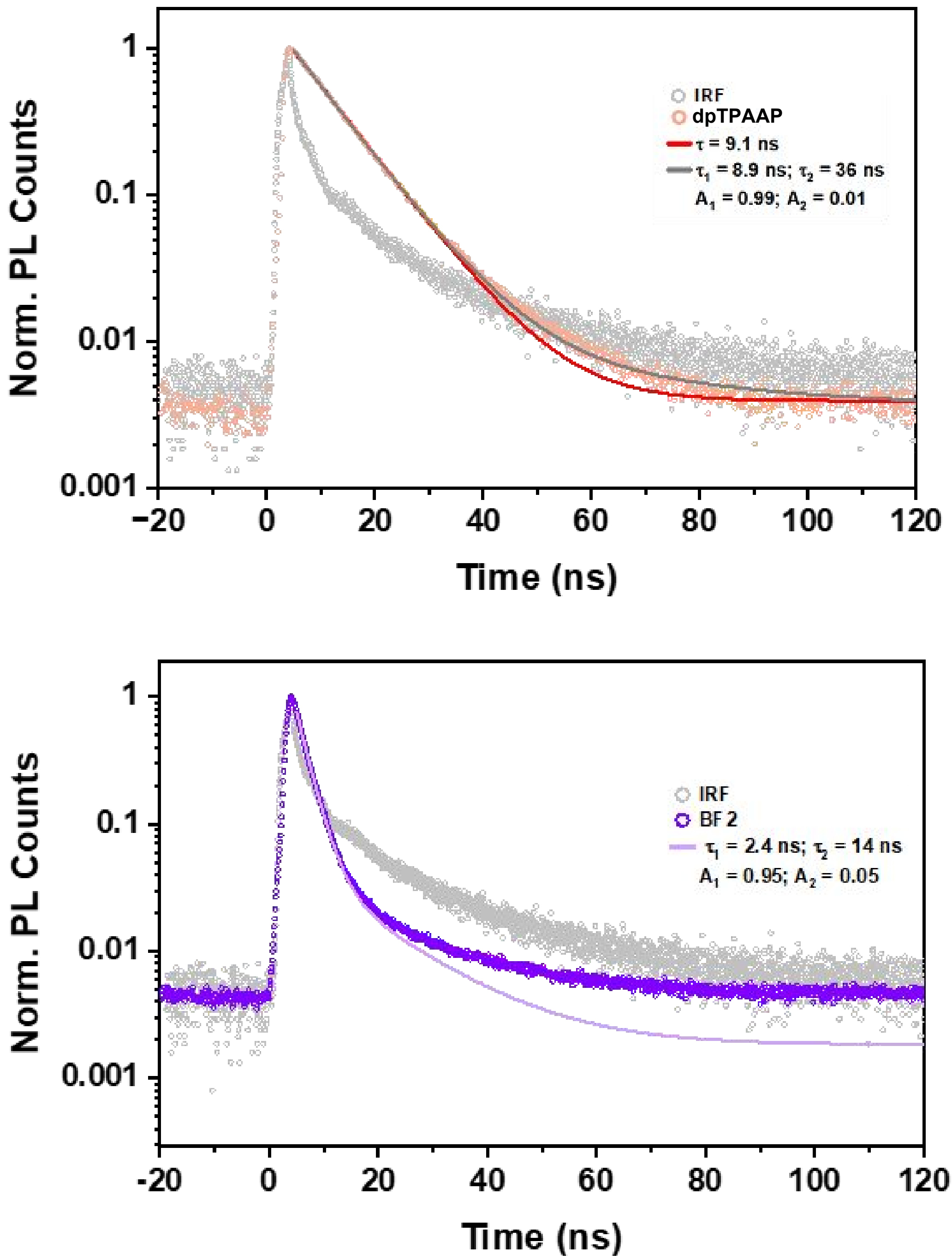

Figure S5. TCSPC traces for the prompt emission of BF2 and dpTPAAP solutions excited at 450 nm.

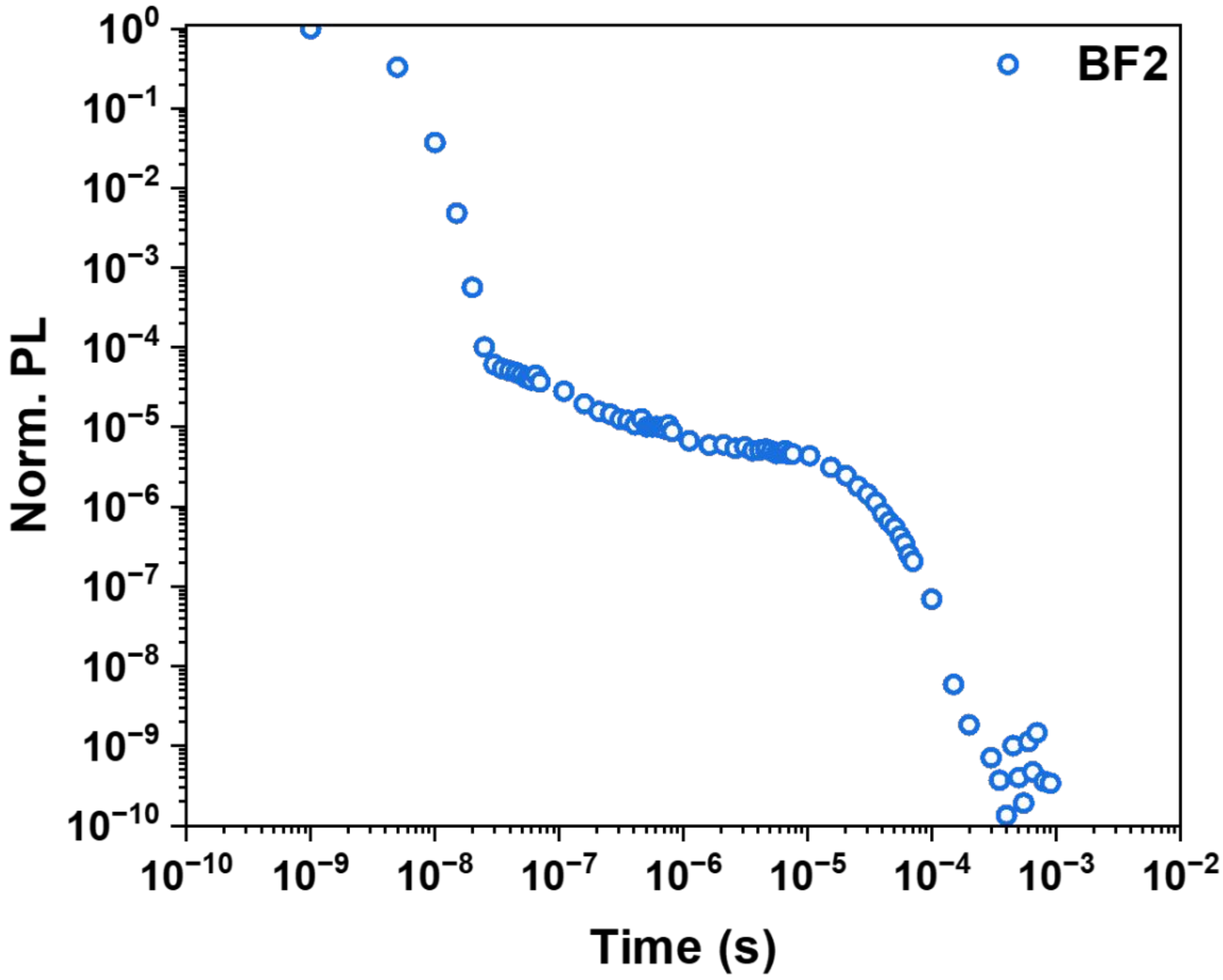

Figure S6. TRPL trace recorded on an ICCD camera detector for a BF2 solution excited at 450 nm. The contribution from delayed PL is approximately 0.1%.

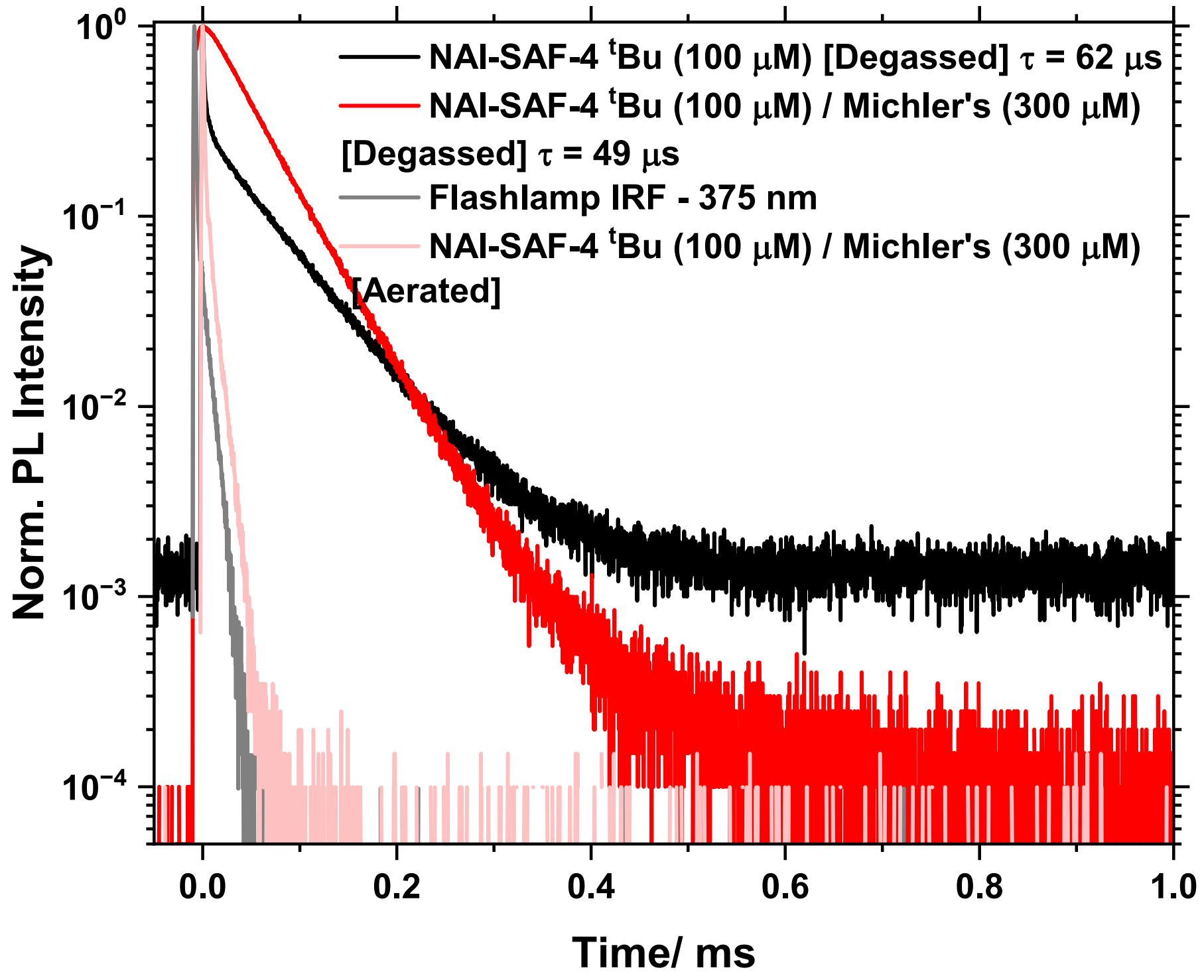

Figure S7. Traces for PL decays recorded using flashlamp MCS for NAI-SAF-4 ᵗBu (100 μM) / Michler's ketone (300 μM) excited at 375 nm.

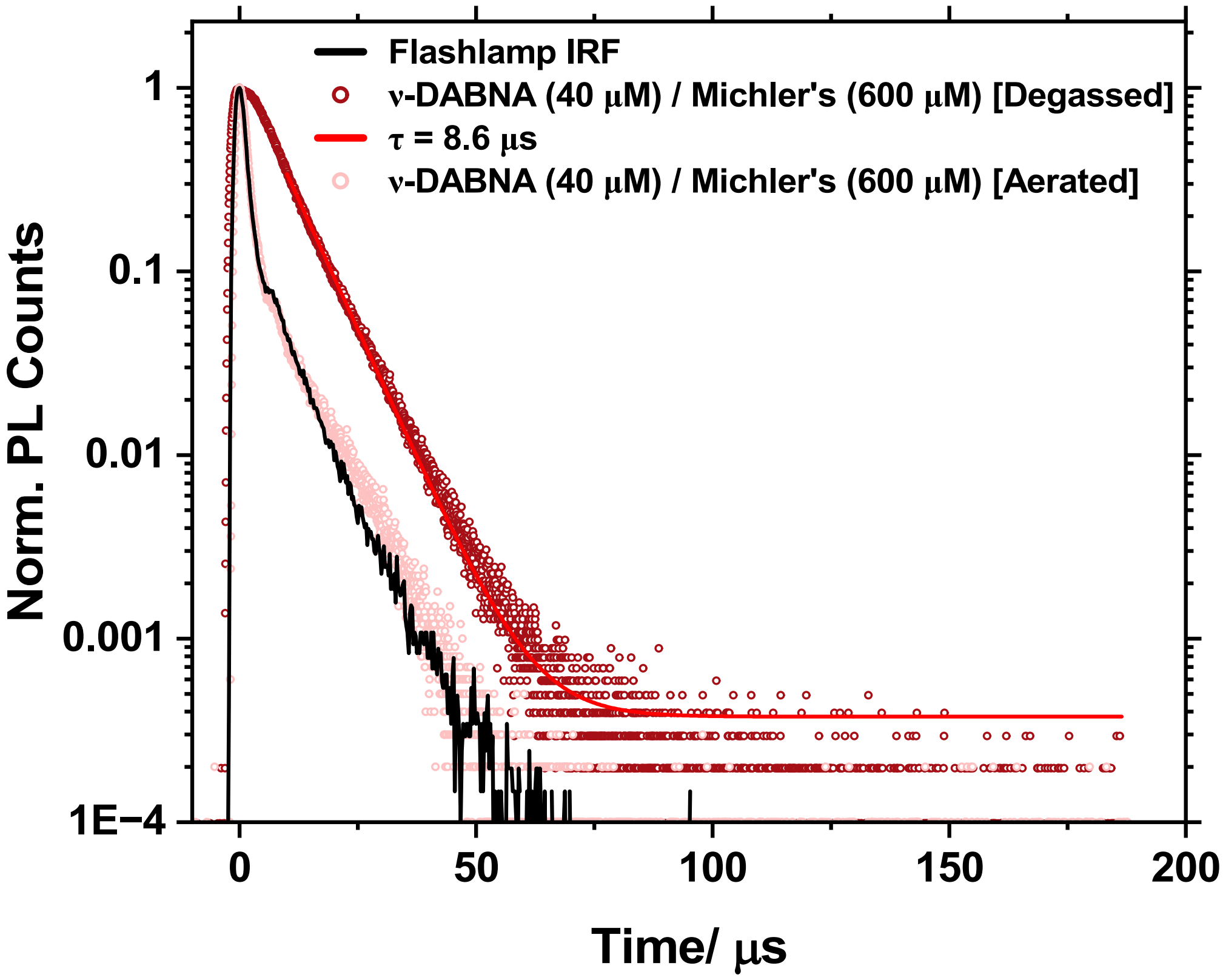

Figure S8. Traces for PL decays recorded using flashlamp MCS for ν-DABNA (40 μM) / Michler's ketone (600 μM) excited at 350 nm.

.

S7. Additional Oxygen Quenching Data

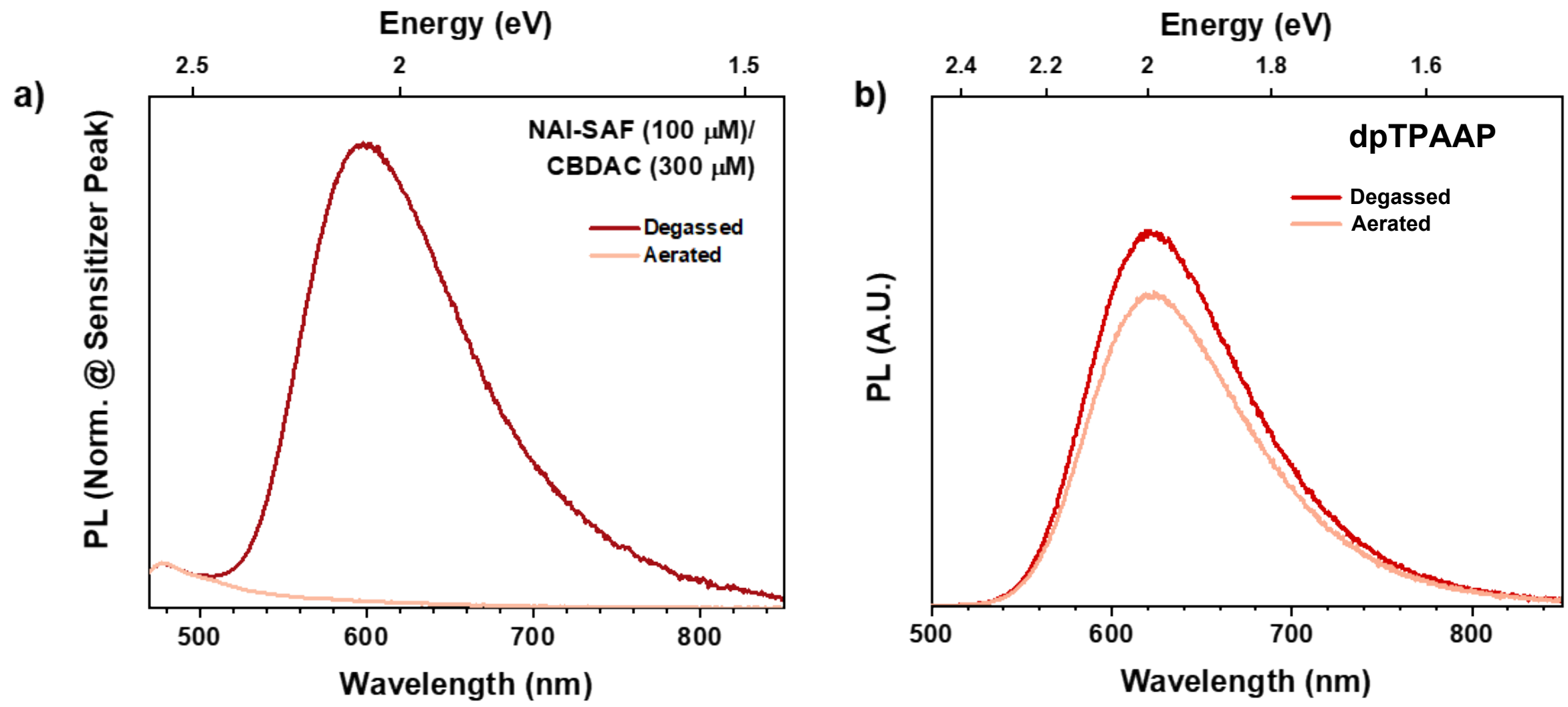

Figure S9. a) Steady state PL for a mixed solution of NAI-SAF 4 ^{t}Bu and CBDAC in toluene when aerated and degassed by argon bubbling. Spectra are normalized to trace CBDAC PL, which shows negligible sensitivity to air. b) Steady state PL for a solution of dpTPAAP in toluene when aerated and degassed by argon bubbling

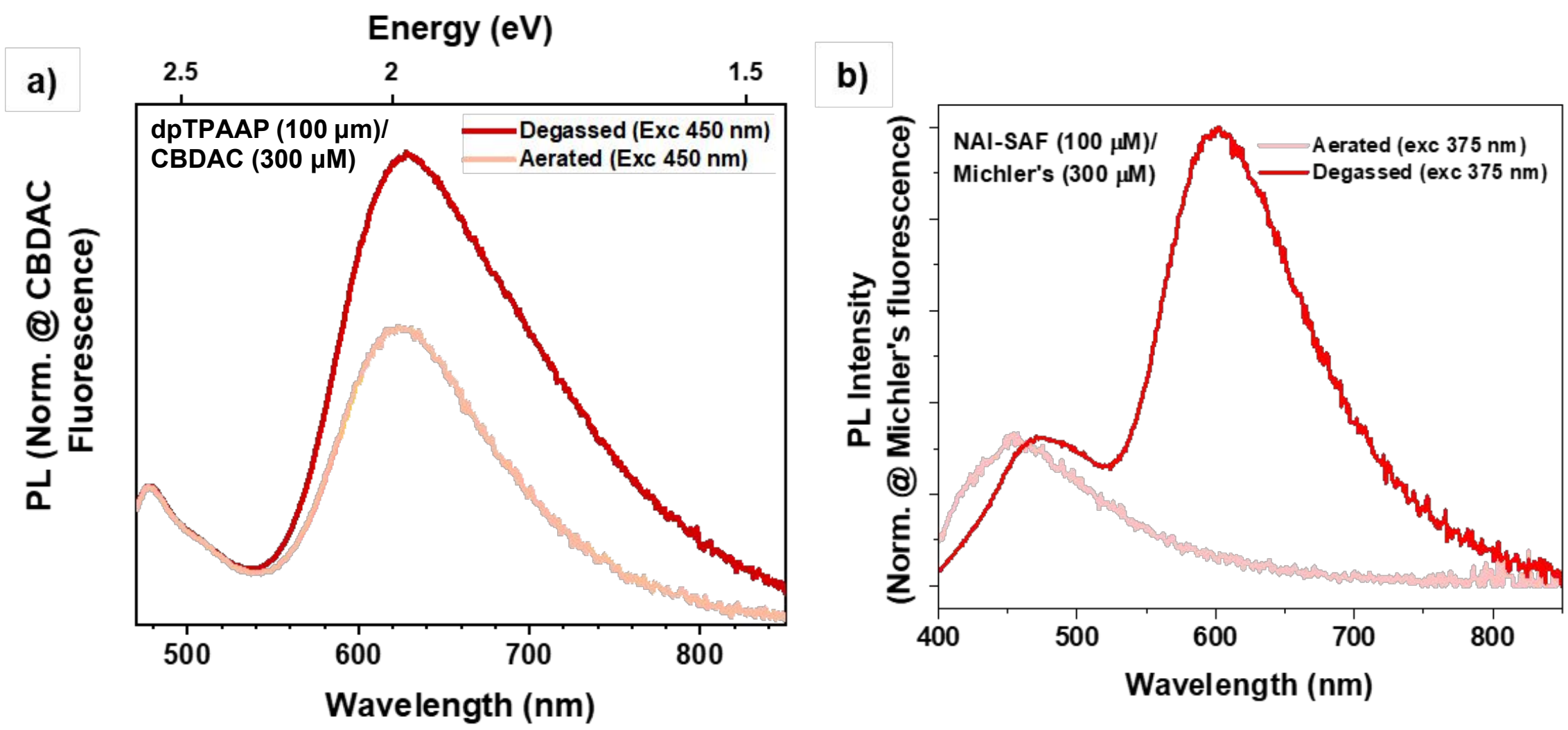

Figure S10. a) Steady state PL for a mixed solution of dpTPAAP and CBDAC in toluene when aerated and degassed by argon bubbling; b) Steady state PL for a mixed solution of NAI-SAF 4 ^{t}Bu and Michler's ketone in toluene when aerated and degassed by argon bubbling. Spectra are normalized to trace sensitizer PL, which shows negligible sensitivity to air.

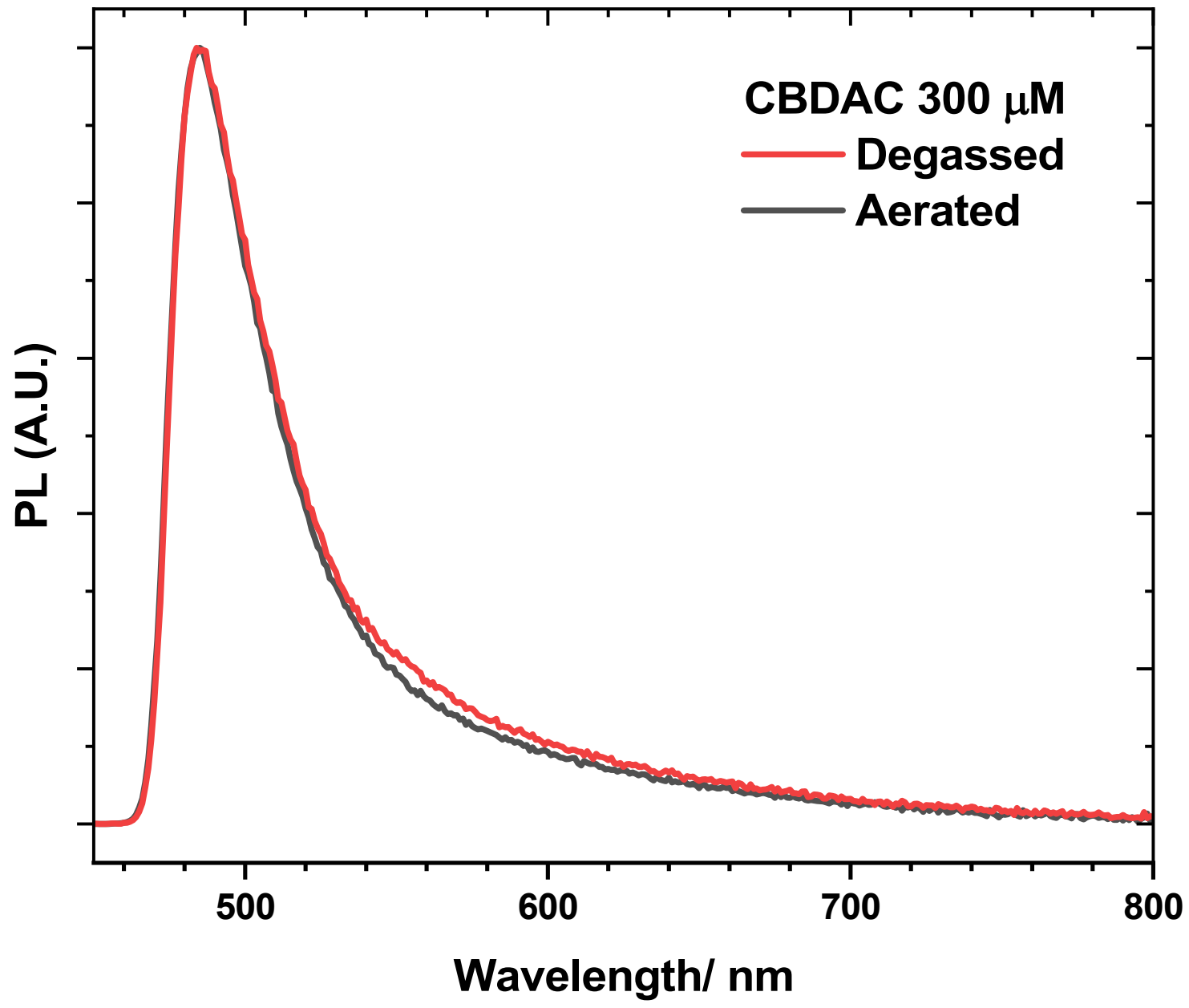

Figure S11. Steady state PL for a solution of CBDAC in toluene when aerated and degassed by argon bubbling.

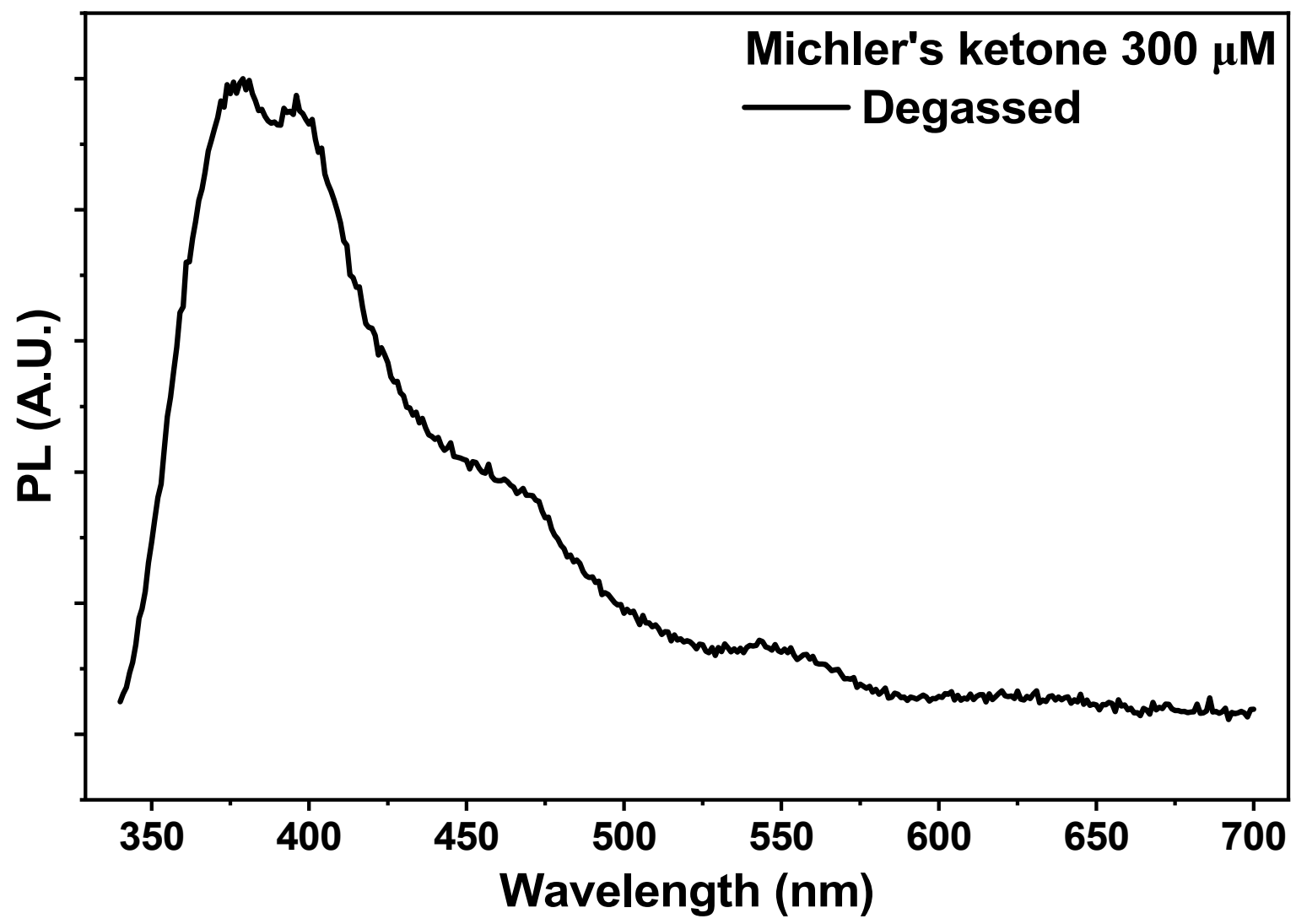

Figure S12. Steady state PL for a solution of Michler's ketone in toluene when degassed by argon bubbling.

S8. Additional Excitation Fluence Data

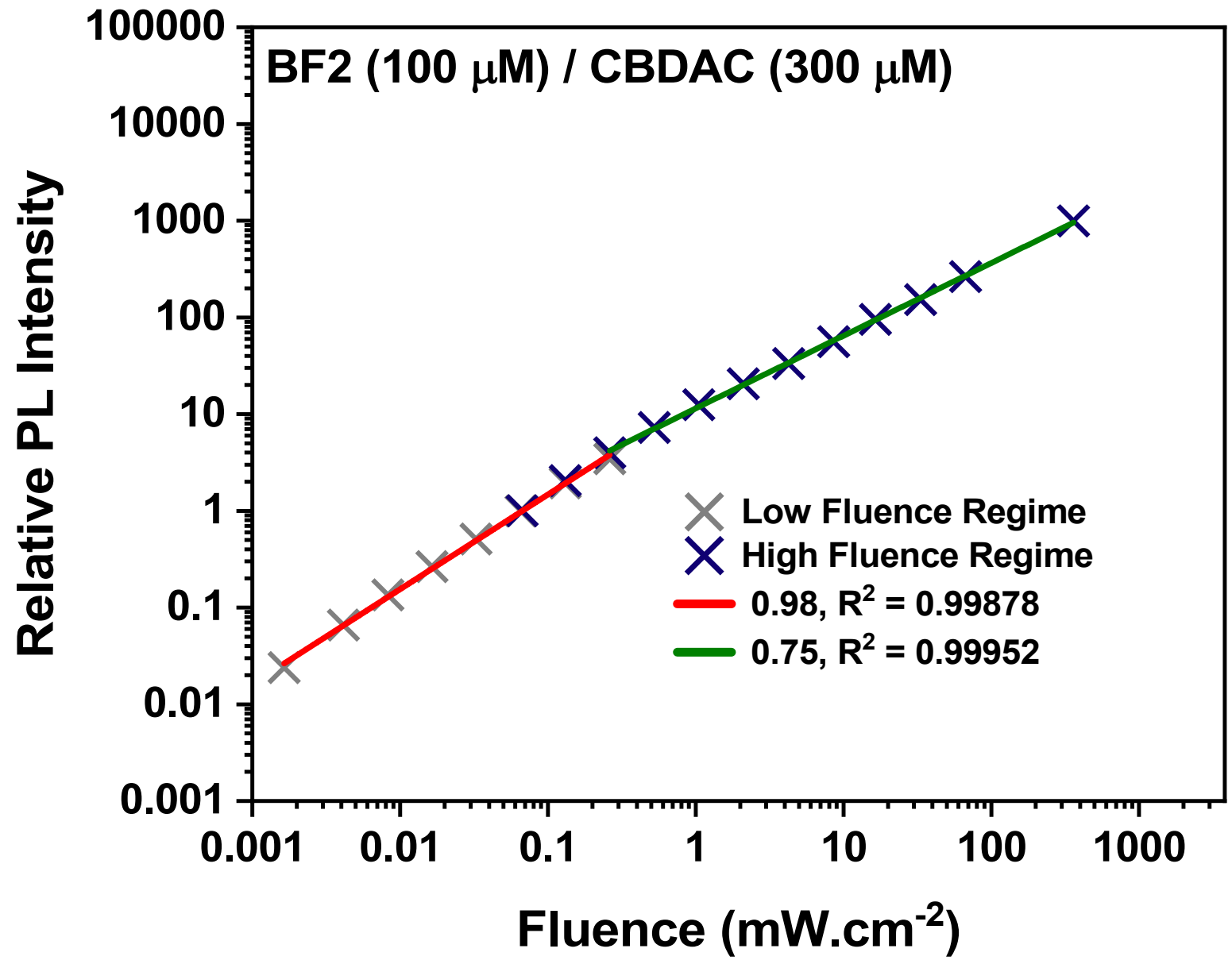

Figure S13. Excitation fluence dependence for a mixed solution of BF2 and CBDAC in toluene.

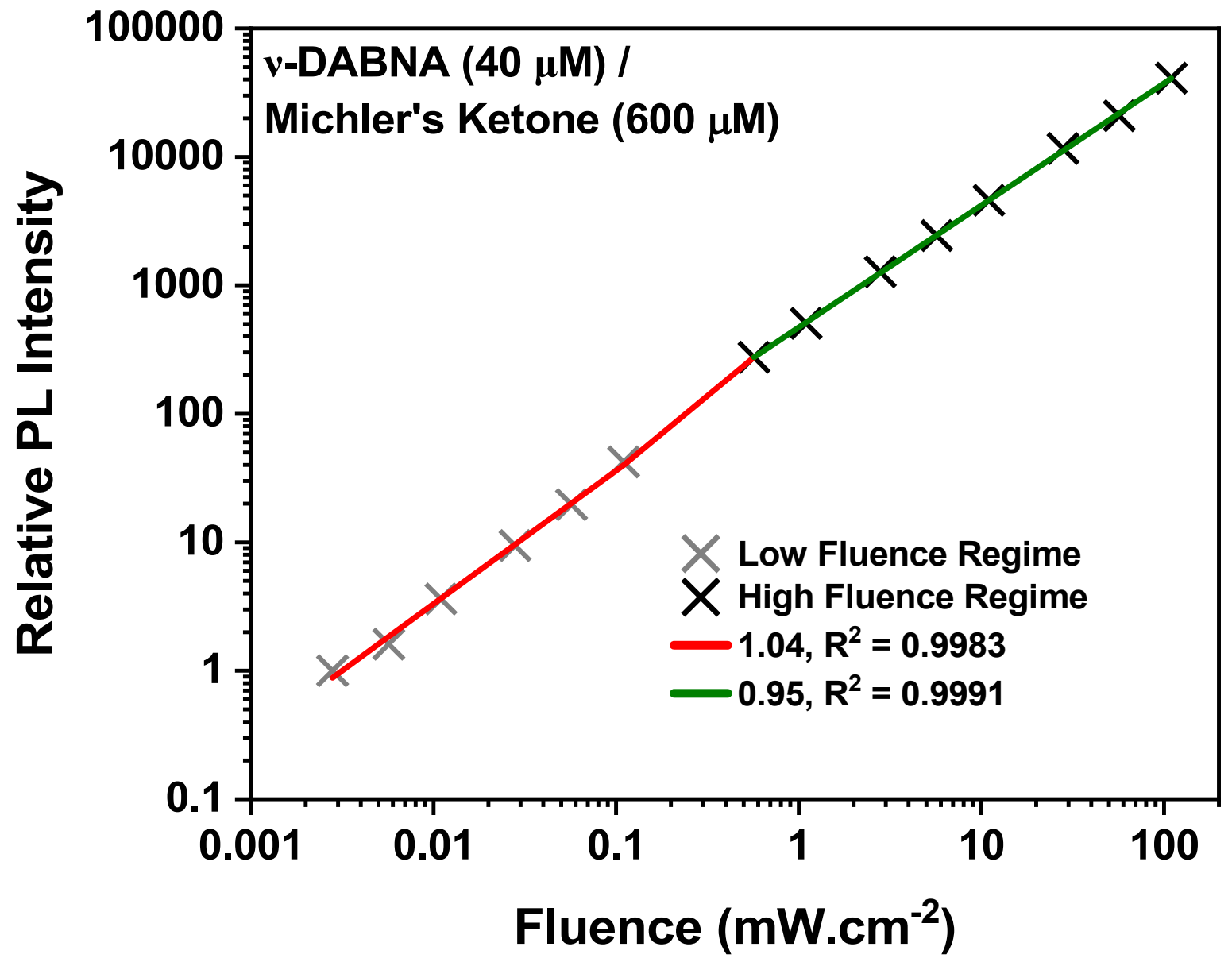

Figure S14. Excitation fluence dependence for a mixed solution of ν-DABNA and Michler's Ketone in toluene.

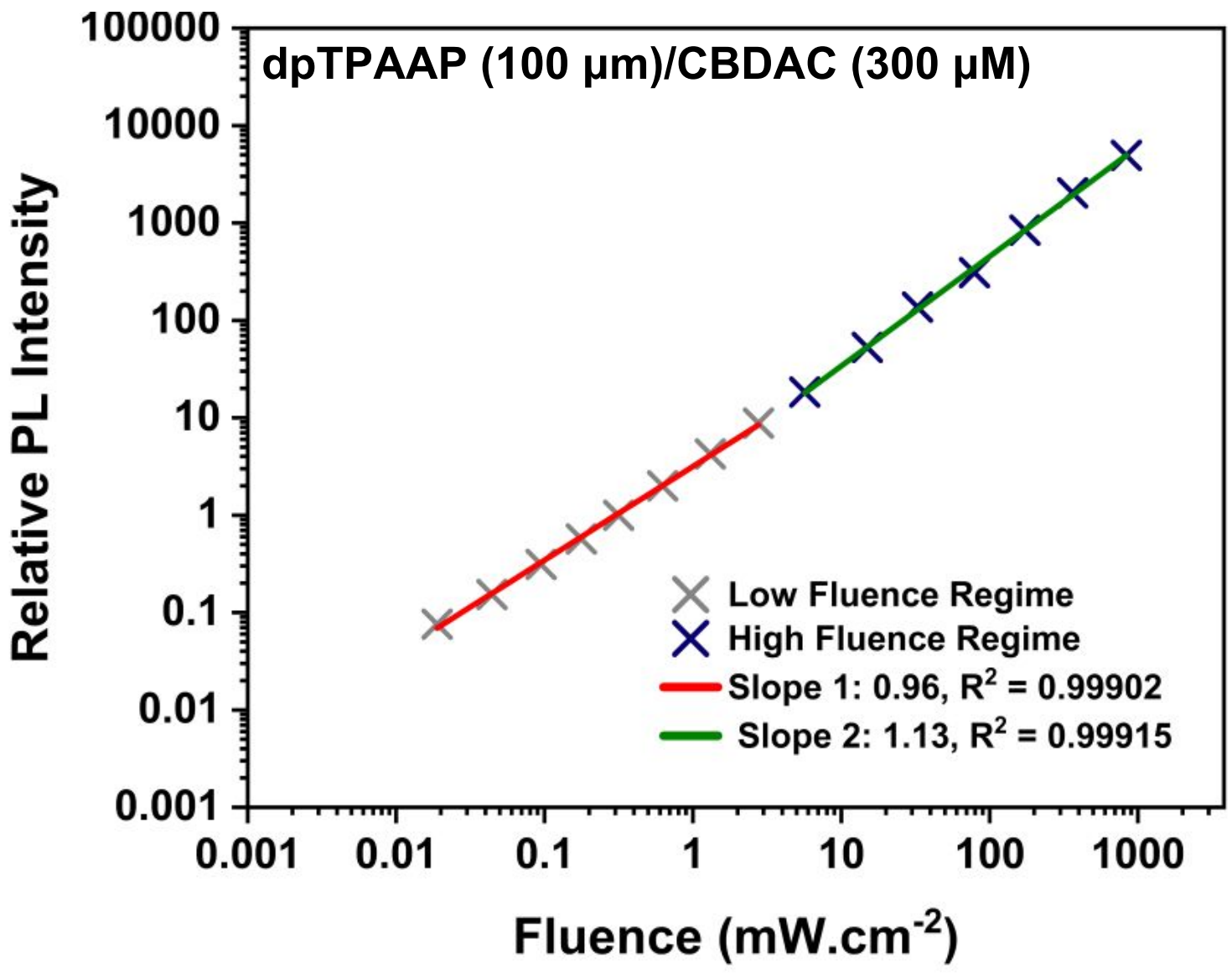

Figure S15. Excitation fluence dependence for a mixed solution of dpTPAAP and CBDAC in toluene.

S9. Computational Chemistry

General

All calculations were carried out with the Gaussian 09 package.[11] Optimized S_0 geometries were determined using B3LYP[12,13] with the 6–31G* basis set.[14,15] All S_0 geometries were true minima based on no imaginary frequencies found. Electronic structure and TD-DFT calculations were carried out on the optimized geometries at TD–PBE0/def2-TZVP.[16,17] The MO diagrams were generated with the aid of Gabedit[18] or Gaussview.

Table S10. TD-DFT data calculated for dpTPAAP at PBE0/def2-TZVP (Ground state geometry, vertical transition).

State	eV	nm	f	D index	Sr	T index	orbitals
T_1	1.828	678.26	0	6.596	0.40178	3.136	H-L:0.706; H2-L:0.178
S_1	2.0908	592.99	0.2786	9.105	0.25827	6.118	H-L: 0.988
T_2	2.2824	543.22	0	3.564	0.63287	-0.259	H3-L:0.408; H2-L:0.264
T_3	2.3983	516.97	0	2.092	0.65344	-2.091	H6-L:0.349; H2-L:0.135
T_4	2.5688	482.65	0	8.244	0.39326	4.450	H-L1:0.771; H-L4:0.049

PBE0/def2-TZVP

ΔE_{ST} vertical (PBE0/def2-TZVP) = 0.2628 eV

ΔE_{S1-T2} vertical (PBE0/def2-TZVP) = −0.1916 eV

SOCME$_{S1-Tn}$

T_1 = 0.27 cm^{-1}

T_2 = 0.17 cm^{-1}

Adiabatic

ΔE_{ST} adiabatic (PBE0/def2-TZVP) = 0.2031 eV

Hole-electron analysis

Hole-electron analysis was conducted using Multiwfn.[19–21] . In electron-hole analysis the S_r index is a measure to characterize the overlapping extent of holes and electrons by integration of the geometric mean of their charge densities over all space, which approaches zero as CT character increases. The D

index is the total magnitude of CT length as measured by the distance between the centres of mass of electrons and holes, hence D typically increases with CT character. The t index represents the difference between the D index and the average spatial extension degree of hole and electron distribution in the CT direction, characterizing the separation degree of holes and electrons in the CT direction. Negative t indices imply that the holes and electrons are not substantially separated due to CT.

It is important to consider all parameters when assigning state character. The S_1 state of dpTPAAP is a HOMO → LUMO transition, with clear CT character – a small S_r index, a large D index and large positive T index. An important aspect of CAT-5 is that the T_1 is different in character to the S_1. Rather than a classical CT state, T_1 is a hybrid CT state with some LE character. As well as HOMO → LUMO character (71%), there is also some HOMO−2 → LUMO contribution (18%). The HOMO−2 orbital clearly has some localisation onto the electron acceptor as well as on the electron donor (Figure S8). The mixed character is evident from the electron-hole analysis. The S_r, D and T indices still correlate with CT character for T_1, but to less extreme an extent in comparison to S_1. Owing to the mixed character of T_1, S_1 and T_1 have a degree of difference in both character and spatial distribution. Hence, a $S_1 \leftrightarrow T_1$ spin-flip should be promoted according to El-Sayed's rule, in comparison to two states of pure HOMO → LUMO CT character. This is reflected in the reasonable S_1-T_1 SOCME[22] value of 0.27 cm^{-1}. Hence RISC is promoted, and when the T_1 state of dpTPAAP is populated via sensitization TADF is possible in solution, despite the experimental $\Delta E_{ST} = 0.24$ eV.

From Fermi's Gold Rule:

$$k_{RISC} \propto \frac{2\pi}{\hbar} \left| \langle S_1 \,|\, H_{SO} \,|\, T_1 \rangle \right|^2 \times \rho(\Delta E_{ST})$$

Where $\langle S_1 | H_{SO} | T_1 \rangle$ is the SOCME, and $\rho(\Delta E_{ST})$ is the Franck-Condon weighted density reflecting the vibration contribution and related ΔE_{ST}.

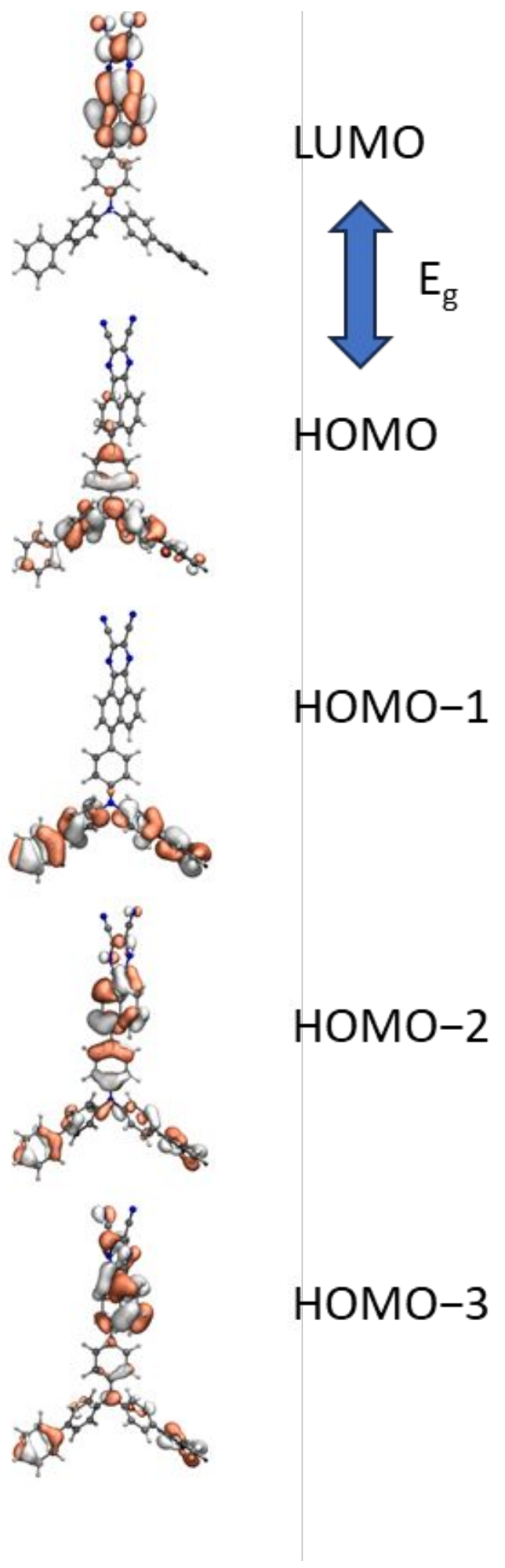

Figure S16. Plots to show the localisation of orbitals pertinent to the lowest energy singlet and triplet excited states of dpTPAAP.

S10. Kinetic Modelling

Kinetic modelling was done using MATLAB. The differential equations describing the sensitized system used for the modelling are described below:

$$\frac{d[^3Sen^*]}{dt} = -\, k_{TET}[^3Sen^*][^1TADF] - k_{Tsens}[^3Sen^*] \qquad \text{(Eq. S1)}$$

$$\frac{d[^3TADF^*]}{dt} = -(k_T + k_{RISC})[^3TADF^*] + k_{ISC}[^1TADF^*] + k_{TET}[^3Sen^*][^1TADF] \qquad \text{(Eq. S2)}$$

$$\frac{d[^1TADF^*]}{dt} = -(k_r + k_{nr} + k_{ISC})[^1TADF^*] + k_{RISC}[^3TADF^*] \qquad \text{(Eq. S3)}$$

$$\frac{d[^1TADF]}{dt} = (k_r + k_{nr})[^1TADF^*] + k_T[^3TADF^*] \; - k_{TET}[^3Sen^*][^1TADF] \qquad \text{(Eq. S4)}$$

$$\frac{d[^1Sen]}{dt} = k_{TET}[^3Sen^*][^1TADF] + k_{Tsens}[^3Sen^*] \qquad \text{(Eq. S5)}$$

Where Sen refers to sensitizer, TADF to the TADF material, [..] denotes concentration, the asterix denotes excited states and superscripts denotes singlet (1) and triplet (3) spin states. k_T and k_{Tsens} are the triplet decay (radiative and non-radiative) rate constant for the TADF and sensitizer triplet states, respectively. k_{TET} is the bimolecular triplet energy transfer rate constant, k_{ISC} and k_{RISC} are the inter system crossing and reverse inter system crossing rate constants for the TADF material. k_r and k_{nr} are the radiative and non radiative decay rate constants from the singlet excited state of the TADF material.

We use the built in MATLAB function ode23s to solve the set of differential equations with an initial sensitizer triplet concentration set to 1 μM. The time range is divided into smaller ranges to ensure smoot solutions using ode23s. The same set of equations can be solved for the direct excitation of the TADF material by setting either the triplet energy transfer rate to 0, or fixing the initial concentration of sensitizer to 0. Rate constants are estimated from complimentary experiments and manually adjusted to yield satisfactory description of the experimental data. The triplet energy transfer rate was set to a diffusion limited value of $1 \times 10^9 M^{-1}s^{-1}$ which yielded an adequate agreement with the experimental data, Figure S15. Other rate constants used for NAI-SAF ^{4}tBu and BF2 are summarized in table S11 below.

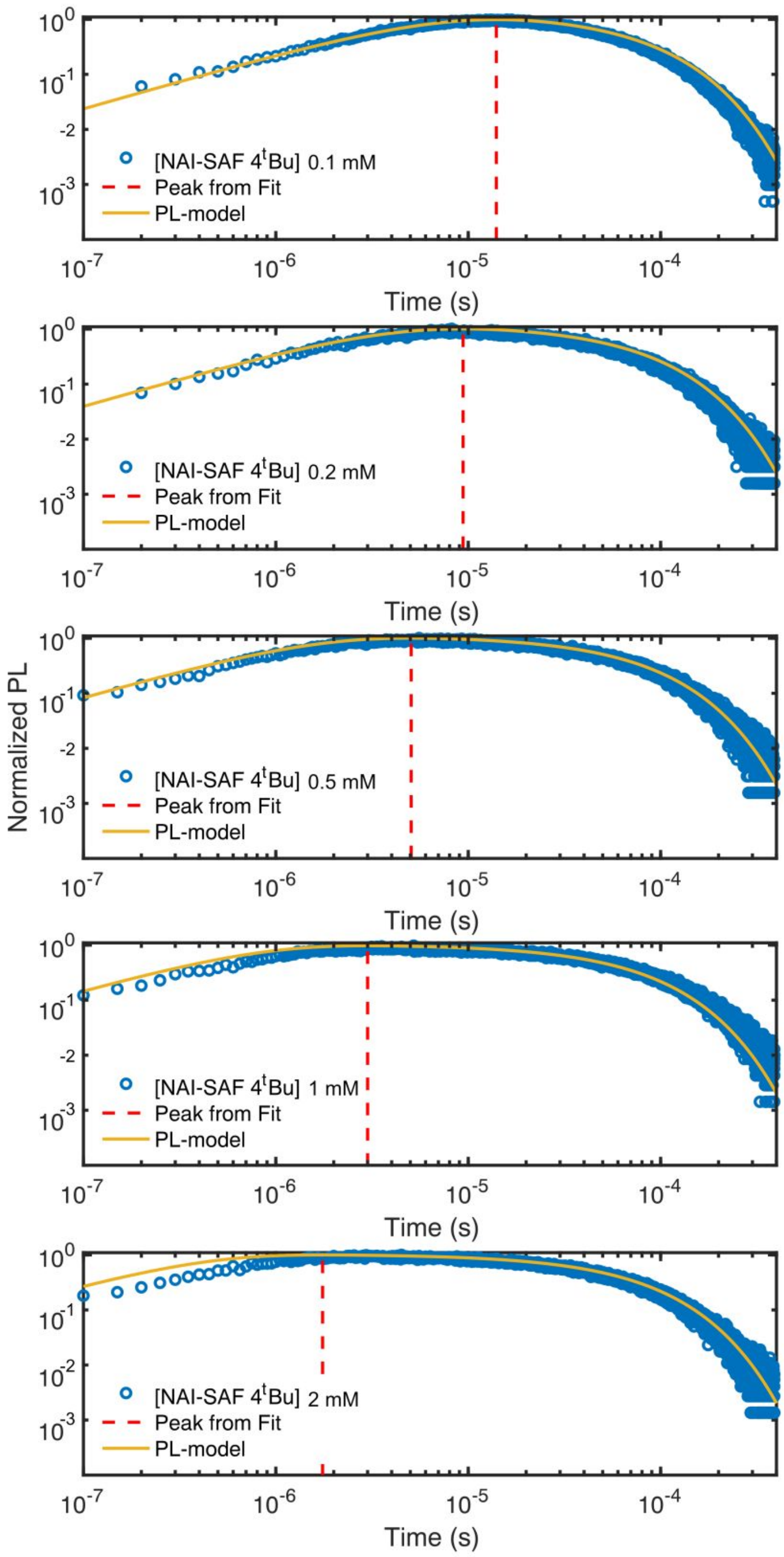

Figure S17. Experimental data and model PL kinetics for a mixed solution of CBAC (300 μM) / NAI-SAF-4^tBu where the TADF analyte concentration was screened.

Table S11. Rate constants used to model TADF and sensitized TADF behaviour.

**Rate constant**	**NAI-SAF 4 ᵗBu / CBDAC**	**BF2 / CBDAC**	**Varying k_{ISC} (Figure S17)**
k_T	$6.7 \times 10^3 s^{-1}$	$1.0 \times 10^2 s^{-1}$	$2.5 \times 10^3 s^{-1}$
k_r	$3.0 \times 10^6 s^{-1}$	$3.5 \times 10^8 s^{-1}$	$1.0 \times 10^8 s^{-1}$
k_{nr}	$5.9 \times 10^6 s^{-1}$	$4.0 \times 10^7 s^{-1}$	$5.9 \times 10^6 s^{-1}$
k_{ISC}	$9.1 \times 10^7 s^{-1}$	$1.3 \times 10^7 s^{-1}$	$1 \times 10^3 - 1 \times 10^9 s^{-1}$
k_{RISC}	$1.0 \times 10^5 s^{-1}$	$1.4 \times 10^3 s^{-1}$	$k_{ISC} e^{-\frac{\Delta E}{k_b T}}$
k_{TET}	$1.0 \times 10^9 M^{-1} s^{-1}$	1.0 $\times$ 1	$1.0 \times 10^9 M^{-1} s^{-1}$
k_{Tsens}	$4.5 \times 10^4 s^{-1}$	$4.5 \times 10^4 s^{-1}$	$4.5 \times 10^4 s^{-1}$

We next model the sensitization of BF2 and compare it to the experimental PL decay obtained using a flash-lamp excitation experiment, Figure S16. The model is compared to the direct excitation of only BF2 and the sensitized system. Overall, the model can explain the enhanced TADF signal, however the model does not consider the broad IRF from the flash-lamp and thus predicts a rise of the sensitized delayed PL, as was the case of NAI-SAF 4ᵗBu (Figure S15). Even when accounting for a small fraction of direct excitation of TADF the rise is discernible in the model (Figure S16). Hence, we conclude that the ~2.5% direct excitation of TADF, with the large fraction of prompt PL of BF2 combined with the broad IRF hides the rise of the sensitized delayed PL. Nevertheless, the fraction of delayed PL is significantly enhanced.

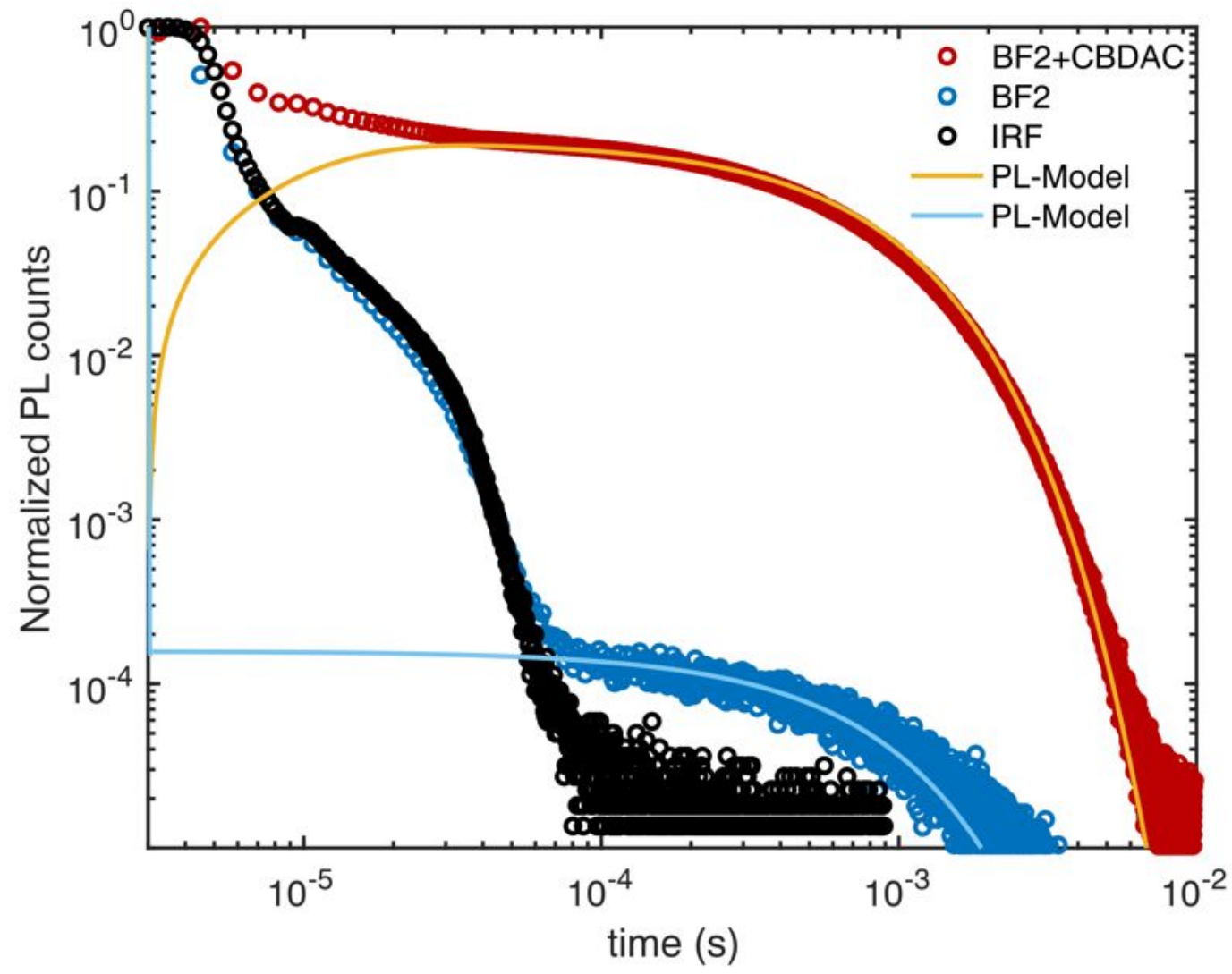

Figure S18. Experimental data (open circles) of BF2 (blue), 100 μM BF2 sensitized by 300 μM CBDAC (red) and model PL kinetics with excitation of BF2 (light blue) and the sensitizer CBDAC (yellow).

With the good agreement between the model and the experimental data of both NAI-SAF 4 tBu and BF2 sensitized TADF behaviour we now use the model to further explore the limits and possibilities of the sensitization method. The challenge of observing TADF with contemporary direct excitation techniques often relates to the amount of prompt PL, and with decreasing ISC rates the prompt component will become larger. It is often hard to distinguish more than 3-4 orders of magnitude weaker delayed PL compared to prompt PL. Hence, we first explore the possible enhancement of TADF materials with varying ISC rates, specifically, as ISC becomes negligible. We use the rate constants specified in Table S11 above. k_{RISC} is calculated from k_{ISC} assuming a constant singlet-triplet gap of 0.1 eV. In Figure S17 a and b we showcase how sensitization enhances the delayed PL intensity and enables detection of TADF also in materials where $\Phi_{ISC} \approx 0$. As emphasized in the main text, it is important to minimize the direct excitation of the TADF material to fully exploit the sensitivity enhancement through sensitization, Figure S17b and S17c illustrates this by comparing 0.5% and 5% direct excitation of the TADF material, respectively. The log-y-axis spans 5-order of magnitude, and in Figure S17a where the direct excitation signal is modelled, only TADF systems with $\Phi_{ISC} > 1\%$ show any delayed signal within these limits. With sensitization (Figure S17b), however, TADF systems with Φ_{ISC} as low as 0.01 and 0.001% fall within the same limits, depending on how selective the excitation of the sensitizer is.

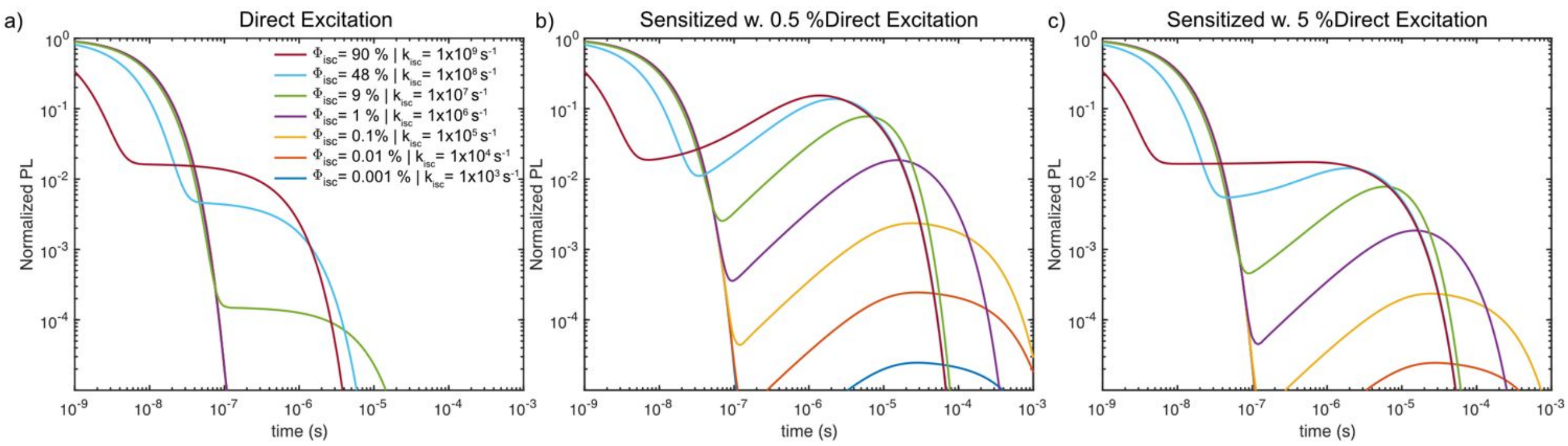

Figure S19. a) model of time-resolved PL emerging from a TADF material upon direct excitation, with decreasing amount of ISC. b) As in a) but with only 0.5% direct excitation and 99.5% excitation of CBDAC sensitizer. c) as in b) but with 5% direct excitation of the sensitizer and 95% excitation of CBDAC sensitizer.

Derivation for expression of delayed lifetime

The rate equations for the non-sensitized TADF system are given by:

$$\frac{d[^3TADF^*]}{dt} = -(k_T + k_{RISC})[^3TADF^*] + k_{ISC}[^1TADF^*] \quad \text{(Eq. S6)}$$

$$\frac{d[^1TADF^*]}{dt} = -(k_r + k_{nr} + k_{ISC})[^1TADF^*] + k_{RISC}[^3TADF^*] \quad \text{(Eq. S7)}$$

Where the rates are defined as above. This two-state system has an analytic solution that has been reported multiple times. The prompt and delayed decay times (τ_p and τ_d, respectively) are given by the inverse of k_p and k_d, the prompt and delayed decay constants, respectively. Furthermore, the decay constants are related to the rate constants in Eq. S6 and S7 by:

$$k_p + k_d = k_r + k_{nr} + k_{ISC} + k_T + k_{RISC} \quad \text{(Eq. S8)}$$

$$k_p k_d = (k_T + k_{RISC})(k_r + k_{nr} + k_{ISC}) - k_{RISC}k_{ISC} \quad \text{(Eq. S9)}$$

For a TADF material with high radiative decay, such as dpTPAAP one can assume $k_r \gg k_{nr} + k_{ISC}$ simplifying the expressions to:

$$k_p + k_d = k_r + k_T + k_{RISC} \quad \text{(Eq. S10)}$$

$$k_p k_d = (k_T + k_{RISC})(k_r) - k_{RISC}k_{ISC} \quad \text{(Eq. S11)}$$

Re-arranging Eq. S10 for an expression for k_p and substituting this expression for k_p in Eq. S11 can after further rearrangement yield an expression for k_{RISC}:

$$k_{RISC} = \frac{k_T k_r + k_d^2 - k_d k_T - k_r k_d}{k_d + k_{ISC} - k_r} \quad \text{(Eq. S12)}$$

Next one can assume that $k_r \gg k_{ISC}$, k_d, $k_d k_T$ and k_d^2, which is valid when radiative decay from the singlet state occurs on the 10s of ns time scale and the delayed decay occurs on the 100s of microsecond timescale. With these assumptions the expression simplifies to:

$$k_{RISC} = \; -k_T + k_d \quad \text{(Eq. S13)}$$

Or expressed as the delayed decay rate constant:

$$k_d = \; k_T + k_{RISC} \quad \text{(Eq. S14)}$$

In literature one often finds the assumption that $k_T \ll k_{RISC}$, meaning that triplet decay mostly occurs through RISC to the singlet, simplifying the expression further where the delayed decay constant is then equal to k_{RISC}. However, in systems where ISC is slow, and consequently the reverse RISC process also rather slow this assumption is not valid. For example, the triplet life-time of many organic non-TADF materials are on the order of 100-1000 μs, the same order as most observed delayed PL decay times in slower RISC TADF materials. Hence, assuming that $k_d = k_{RISC}$ is likely best seen as an upper limit. As TADF materials with larger k_r are developed the prompt PL quantum yield Φ_p will approach unity. Then, using the sensitization technique described in the main text, and minimizing direct excitation of the TADF it could be possible to determine the quantum yield of delayed PL which then can be used to disentangled the intrinsic triplet decay and RISC process by using the following relations:

$$\Phi_d = \Phi_p \Phi_{RISC} \quad \text{(Eq. S15)}$$

$$\Phi_{RISC} = \frac{k_{RISC}}{k_T + k_{RISC}} \quad \text{(Eq. S16)}$$

S11. References

(1) de Mello, J. C.; Wittmannn, H. F.; Friend, R. H. An Improved Experimental Determination of External Photoluminescence Quantum Efficiency. *Adv. Mater.* **1997**, *9* (3), 230–232. https://doi.org/10.1002/adma.200903328.

(2) Pålsson, B. L.; Monkman, A. P. Measurements of Solid-State Photoluminescence Quantum Yields of Films Using a Fluorimeter. *Adv. Mater.* **2002**, *14* (10), 757–758. https://doi.org/10.1002/1521-4095(20020517)14.

(3) Ahn, T. S.; Al-Kaysi, R. O.; Müller, A. M.; Wentz, K. M.; Bardeen, C. J. Self-Absorption Correction for Solid-State Photoluminescence Quantum Yields Obtained from Integrating Sphere Measurements. *Rev. Sci. Instrum.* **2007**, *78* (8), 086105. https://doi.org/10.1063/1.2768926.

(4) Kim, D.-H.; D'Aléo, A.; Chen, X.-K.; Sandanayaka, A. D. S.; Yao, D.; Zhao, L.; Komino, T.; Zaborova, E.; Canard, G.; Tsuchiya, Y.; Choi, E.; Wu, J. W.; Fages, F.; Brédas, J.-L.; Ribierre, J.-C.; Adachi, C. High-Efficiency Electroluminescence and Amplified Spontaneous Emission from a Thermally Activated Delayed Fluorescent Near-Infrared Emitter. *Nat. Photonics* **2018**, *12* (2), 98–104. https://doi.org/10.1038/s41566-017-0087-y.

(5) Xue, J.; Xu, J.; Ren, J.; Liang, Q.; Ou, Q.; Wang, R.; Shuai, Z.; Qiao, J. Intermolecular Charge-Transfer Aggregates Enable High-Efficiency near-Infrared Emissions by Nonadiabatic Coupling Suppression. *Sci. China Chem.* **2021**, *64* (10), 1786–1795. https://doi.org/10.1007/s11426-021-1096-8.

(6) Millington, O.; Montanaro, S.; Leventis, A.; Sharma, A.; Dowland, S. A.; Sawhney, N.; Fallon, K. J.; Zeng, W.; Congrave, D. G.; Musser, A. J.; Rao, A.; Bronstein, H. Soluble Diphenylhexatriene Dimers for Intramolecular Singlet Fission with High Triplet Energy. *J. Am. Chem. Soc.* **2023**, *145* (4), 2499–2510. https://doi.org/10.1021/jacs.2c12060.

(7) Fujisawa, K.; Beuchat, C.; Humbert-Droz, M.; Wilson, A.; Wesolowski, T. A.; Mareda, J.; Sakai, N.; Matile, S. Anion-π and Cation-π Interactions on the Same Surface. *Angew. Chemie - Int. Ed.* **2014**, *53* (42), 11266–11269. https://doi.org/10.1002/anie.201407161.

(8) Specht, D. P.; Martic, P. A.; Farid, S. Ketocoumarins. A New Class of Triplet Sensitizers. *Tetrahedron* **1982**, *38* (9), 1203–1211. https://doi.org/10.1016/0040-4020(82)85104-1.

(9) Park, I. S.; Yang, M.; Shibata, H.; Amanokura, N.; Yasuda, T. Achieving Ultimate Narrowband and Ultrapure Blue Organic Light-Emitting Diodes Based on Polycyclo-Heteraborin Multi-

Resonance Delayed-Fluorescence Emitters. *Adv. Mater.* **2022**, *34* (9), 2107951. https://doi.org/10.1002/adma.202107951.

(10) Pan, K. C.; Li, S. W.; Ho, Y. Y.; Shiu, Y. J.; Tsai, W. L.; Jiao, M.; Lee, W. K.; Wu, C. C.; Chung, C. L.; Chatterjee, T.; Li, Y. S.; Wong, K. T.; Hu, H. C.; Chen, C. C.; Lee, M. T. Efficient and Tunable Thermally Activated Delayed Fluorescence Emitters Having Orientation-Adjustable CN-Substituted Pyridine and Pyrimidine Acceptor Units. *Adv. Funct. Mater.* **2016**, *26* (42), 7560–7571. https://doi.org/10.1002/adfm.201602501.

(11) Frisch, M. J.; Trucks, G.W.; Schlegel, H. B.; Scuseria, G. E.; Robb, M. A.; Cheeseman, J. R.; Scalmani, G.; Barone, V.;Mennucci, B.; Petersson, G. A.; Nakatsuji, H.; Caricato, M.; Li, X.; Hratchian, H. P.; Izmaylov, A. F.; Bloino, J.; Zheng, G.; Sonnenber, D. J. Gaussian 09. *Gaussian, Inc. Wallingford CT* **2009**, 2–3. https://doi.org/111.

(12) Becke, A. D. Density-Functional Thermochemistry. III. The Role of Exact Exchange. *J. Chem. Phys.* **1993**, *98* (7), 5648. https://doi.org/10.1063/1.464913.

(13) Lee, C.; Yang, W.; Parr, R. G. Development of the Colle-Salvetti Correlation-Energy Formula into a Functional of the Electron Density. *Phys. Rev. B* **1988**, *37* (2), 785–789. https://doi.org/10.1103/PhysRevB.37.785.

(14) Petersson, G. A.; Al-Laham, M. A. A Complete Basis Set Model Chemistry. II. Open-Shell Systems and the Total Energies of the First-Row Atoms. *J. Chem. Phys.* **1991**, *94* (9), 6081–6090. https://doi.org/10.1063/1.455064.

(15) Petersson, G. A.; Bennett, A.; Tensfeldt, T. G.; Al-Laham, M. A.; Shirley, W. A. A Complete Basis Set Model Chemistry. I. The Total Energies of Closed-Shell Atoms and Hydrides of the First-Row Elements. *J. Chem. Phys.* **1988**, *89* (4), 2193–2218. https://doi.org/10.1017/CBO9781107415324.004.

(16) Moral, M.; Muccioli, L.; Son, W.; Olivier, Y.; Sancho-Garcia, J. C. Theoretical Rationalization of the Singlet − Triplet Gap in OLEDs Materials : Impact of Charge-Transfer Character. *J. Chem. Theory Comput.* **2015**, *11*, 168–177. https://doi.org/10.1021/ct500957s.

(17) Zeng, W.; Gong, S.; Zhong, C.; Yang, C. Prediction of Oscillator Strength and Transition Dipole Moments with the Nuclear Ensemble Approach for Thermally Activated Delayed Fluorescence Emitters. *J. Phys. Chem. C* **2019**, *123*, 10081–10086. https://doi.org/10.1021/acs.jpcc.9b02376.

(18) Allouche, A.-R. Gabedit: A Graphical User Interface For Computational Chemistry Softwares. *J. Comput. Chem.* **2011**, *32* (1), 174–182. https://doi.org/10.1002/jcc.21600.

(19) Lu, T.; Chen, F. Multiwfn : A Multifunctional Wavefunction Analyzer. *J. Comput. Chem.* **2011**,

33, 580–592. https://doi.org/10.1002/jcc.22885.

(20) Zeng, W.; Zhou, T.; Ning, W.; Zhong, C.; He, J.; Gong, S.; Xie, G.; Yang, C. Realizing 22.5% External Quantum Efficiency for Solution-Processed Thermally Activated Delayed-Fluorescence OLEDs with Red Emission at 622 Nm via a Synergistic Strategy of Molecular Engineering and Host Selection. *Adv. Mater.* **2019**, 1901404. https://doi.org/10.1002/adma.201901404.

(21) Zhuang, W.; Wang, S.; Tao, Q.; Ma, W.; Berggren, M.; Fabiano, S.; Zhu, W.; Wang, E. Synthesis and Electronic Properties of Diketopyrrolopyrrole-Based Polymers with and without Ring-Fusion. *Macromolecules* **2021**, *54* (2), 970–980. https://doi.org/10.1021/acs.macromol.0c02326.

(22) Gao, X.; Bai, S.; Fazzi, D.; Niehaus, T.; Barbatti, M.; Thiel, W. Evaluation of Spin-Orbit Couplings with Linear-Response Time-Dependent Density Functional Methods. *J. Chem. Theory Comput.* **2017**, *13* (2), 515–524. https://doi.org/10.1021/acs.jctc.6b00915.
